# Garlic oil improves small intestinal motility in experimentally induced type II diabetes mellitus in female Wistar rats

**DOI:** 10.1371/journal.pone.0301621

**Published:** 2024-04-17

**Authors:** Nermine K. M. Saleh, Abd El-Hamid A. Mohamed, Manal H. Moussa, Yasmin Assal, Noha N. Lasheen

**Affiliations:** 1 Faculty of Medicine, Ain Shams University, Cairo, Egypt; 2 Faculty of Medicine, Galala University, Suez, Egypt; Suez Canal University, EGYPT

## Abstract

Diabetes mellitus adversely affects the contractile ability of the small intestine. However, there is a paucity of studies investigating the impact of garlic oil on small intestinal motility. This study aimed to evaluate the potential beneficial effects of garlic oil on type 2 diabetes mellitus in rats. Thirty-six adult female Wistar rats (n = 36) were divided into four groups: control, non-diabetic rats supplemented with garlic oil, diabetic rats, and diabetic rats treated with garlic oil. The rats were anesthetized using pentobarbitone (40 mg/kg BW); various motility parameters and oxidative markers were determined in small intestinal segments. Measurements were taken for naso-anal length, waist circumference, fasting blood glucose level (FBG), and plasma insulin level. Compared to the control group, the diabetic rats exhibited a reduction in the average force of contraction and motility index in all small intestinal segments. Furthermore, the rats exhibited a reduction in the average duration of muscle contraction only in the jejunum. The rats also exhibited hyperglycemia, insulin resistance, significant oxidative stress, and obesity. This was proven by changes in motility parameters, fasting blood glucose levels, HOMA-IR values, intestinal MDA levels, and waist circumference. The non-diabetic rats supplemented with garlic oil also exhibited a decrease in the average force of contraction and motility index in all small intestinal segments, despite having consistently higher Lee index and waist circumference values. However, the diabetic rats treated with garlic oil demonstrated improved small intestinal motility in nearly all small intestinal segments and a reduction in oxidative stress. In conclusion, rats with diabetes mellitus experienced a decrease in small intestinal motility, which is primarily driven by oxidative stress. Normal rats administered with garlic oil supplements exhibited similar effects. In contrast, garlic oil treatment in diabetic rats led to enhanced small intestinal motility and a notable anti-hyperglycemic effect, which can be attributed to the potent antioxidant properties of garlic oil.

## Introduction

Type 2 diabetes mellitus (DM) arises from an imbalance between insulin sensitivity and insulin secretion. This condition is characterized by impaired insulin action and altered glucose levels in various tissues, including skeletal muscle, adipose tissue, and the liver. To compensate for these abnormalities and maintain normal glucose levels, individuals at high risk of developing DM experience hyperinsulinemia. However, as β-cell function declines in these individuals, impaired glucose tolerance and overt DM may manifest [1]. Consequently, the incidence of type 2 DM has been associated with lifestyle patterns that contribute to obesity [2]. Both type 1 and type 2 diabetic patients have demonstrated a link between hyperglycemia and the pathogenesis of microvascular complications [3]. Diabetic autonomic neuropathy, a common complication affecting approximately 20% of all diabetic patients, can remain undiagnosed despite its impact on various systems, including the gastrointestinal, genitourinary, and cardiovascular systems. Gastrointestinal disturbances associated with diabetic autonomic neuropathy include esophageal dysfunction, gastroparesis, constipation, diarrhea, and fecal incontinence [4]. Consequently, any section of the gastrointestinal tract may be affected in individuals with diabetes. Small intestinal dysfunction appears to be more prevalent in DM compared to esophageal or gastric dysfunction and is often associated with more severe symptoms [5].

The small intestine of diabetic patients undergoes structural and functional changes that contribute to motility impairments, delayed transit time, and disruptions in both secretory and absorptive functions [6]. Furthermore, DM can deplete the neurons of the small intestine and reduce its stimulatory actions [7].

Chronic hyperglycemia in diabetic patients is believed to induce oxidative stress, which plays a crucial role in cellular injury by promoting the generation of free radicals. These free radicals can cause damage to cell membranes and DNA. The increased production and/or decreased destruction of reactive oxygen species (ROS) contribute to elevated levels of oxidative stress in DM. Non-enzymatic and enzymatic antioxidants, such as catalase (CAT), glutathione peroxidase (GSH-Px), and superoxide dismutase (SOD), are involved in combating ROS. The levels of these antioxidant enzymes critically influence tissue susceptibility to oxidative stress and are associated with the development of complications in DM. Notably, β islet cells, which are affected in DM, possess low levels of intrinsic antioxidant defenses [8, 9].

On the other hand, garlic is a widely consumed food with well-recognized medicinal properties dating back to ancient times [10]. Garlic oil (*Allium sativum L*.) is a rich source of sulfur-containing compounds, particularly alliin. Volatile compounds such as allicin and lipid-soluble sulfur compounds like diallyl disulfide contribute to the characteristic odor, taste, and biological and therapeutic properties of Allium sativum L. [11].

Allium sativum L. exhibits significant antidiabetic and therapeutic properties. Garlic oil can lower blood sugar levels by stimulating pancreatic insulin synthesis or positively influencing insulin receptors [11]. However, limited information is available regarding the effects of garlic or its derivatives on gastrointestinal function in diabetes mellitus.

## Aim of the work

This study investigated the effects of garlic oil treatment on diabetic rats’ small intestinal motility and demonstrated its possible underlying mechanisms.

## Materials and methods

### Experimental protocol

#### Animals

This study was conducted on 36 adult female Wistar rats weighing 150–180 g at the beginning, obtained from an animal farm in Helwan, Cairo, Egypt. The rats were housed in the Animal House of the Physiology Department, Faculty of Medicine, Ain Shams University, under standard conditions of boarding and feeding. They had free access to water throughout the study period. A 7-day acclimatization period was provided, during which the rats were given regular normal diets consisting of milk, bread, and vegetables daily at 8 a.m.

All animal experiments were performed in accordance with the National Institutes of Health guide for the care and use of Laboratory animals (National Research Council, 2010). Ethical approval was obtained from the Ain Shams Faculty of Medicine Ethical Committee, this study was a thesis for a Master’s Degree in Medical Physiology, Faculty of Medicine, Ain Shams University. The study follows the ARRIVE guidelines.

At the end of the experiment, the rats were euthanized with an anesthesia overdose, and the animal remains were disposed of by incineration.

The rats included in the study were randomly allocated into the following groups:

Group I: Control group (C, n = 9): Rats were fed a control diet and received a single intraperitoneal injection of saline (equivalent to the dose of streptozotocin) and then received distilled water by gavage (equivalent to the dose of garlic oil).
Group II: Garlic Oil Supplemented Non-Diabetic group (GO, n = 9): Rats were fed a control diet and supplemented with garlic oil without inducing diabetes mellitus. The dose of garlic oil was 5 ml/kg/day administered by gavage for 2 consecutive weeks [10].
Group III: Diabetic group (D, n = 9): Diabetes was induced in rats through receiving a high-fat diet for 2 weeks followed by a single intraperitoneal injection of Streptozotocin (Sigma Co.) at a dose of 35 mg/kg body weight [12]. After approximately three days, animals with blood glucose levels greater than 300 mg/dL are considered diabetic [13].
Group IV: Garlic Oil-Treated Diabetic group (DG, n = 9): Diabetes was induced by using the same method as the diabetic group and then treated with garlic oil at a daily dose of 5 ml/kg by gavage for 2 consecutive weeks [10].

Garlic oil “Pure” was purchased from: Teyab Misr Garlic Oil, 30ml/bottle. The phytochemicals resemble bioactive organosulfur compounds found in generic garlic oil such as allicin, diallyl sulfide (DAS), diallyl disulfide (DADS), diallyl trisulfide (DATS), E/Z-ajoene, S-allyl-cysteine (SAC), and S-allyl-cysteine sulfoxide (alliin).

The control diet was prepared by mixing 75 g of bread, 123 ml of milk (equivalent to 15 g dry weight), and 10 g of unsalted Karish cheese per 100 g. The high-fat diet used for the diabetic groups was prepared by adding butter to increase the fat content to 16–17%. The composition of the butter used in the study was analyzed according to Holland and Welch [14].

On the day of sacrifice, the rats were weighed after an overnight fast and anesthetized by intraperitoneal injection of Pentobarbitone (El-Gomhoreya Co.) at a dose of 40 mg/kg body weight. The naso-anal length and waist circumference of each rat were measured. Fasting blood glucose (FBG) was measured from a blood drop obtained from the rat tail using the GlucoDr^™^ SuperSensor Test Meter, AGM-2200, Korea. After a midline abdominal incision, blood was collected from the abdominal aorta in two tubes: an EDTA-containing tube for the subsequent determination of HbA1c in whole blood samples, and a heparinized tube for plasma separation. The separated plasma was stored at -80°C for later determination of insulin levels and calculation of homeostasis model assessment of insulin resistance (HOMA-IR).

After blood collection, the different parts of the gastrointestinal tract were identified. Segments of the duodenum, jejunum, and ileum were dissected and rapidly immersed in freshly prepared Tyrode’s solution for motility studies.

Pieces of the different intestinal segments were removed and preserved in parafilm at -80°C for subsequent determination of malondialdehyde (MDA) levels and catalase (CAT) activity. Other pieces of the intestinal segments were preserved in formaldehyde buffer for subsequent histological examination.

#### Intestinal motility studies

Segments of duodenum, jejunum, and ileum, about 1cm long, were suspended separately in an organ tissue bath containing 33 ml of warmed Tyrode’s solution (37°C), continuously bubbled with 95% O2 and 5% CO2. According to **Mohamed et al.** [15], the examined intestinal segment was connected to an isometric force displacement transducer (Biegestab K30, Hugo Sachs Elektronik, March-Hugstetten, Germany). Intestinal motility was recorded on a 2-channel oscillograph (Washington MD2, Bioscience, Seattle, WA, USA) in which the downstroke represents contraction, and the upstroke represents relaxation. The sensitivity of the recording was 0.13. The speed of recording was 10 mm/sec.

The studied intestinal motility parameters included frequency of contraction (the average number of contractions per minute), average duration of contraction (the average duration of a single contraction, in seconds), average force of contraction (the average force of a single contraction, in grams), and the modified motility index (which was calculated by multiplying the contraction force times the average duration of each contraction in one minute, in gm. min) [16].

Body mass index (BMI) and Lee index were calculated according to **Bernardis** [17], using the following equations:

$$BMI=\frac{Body\;Weight\;(g)}{{Nasoanal\;length}^{2}\;({cm}^{2})}$$

$$Lee\;index=\frac{\sqrt[{3}]{Body\;weight\;(g)}}{Nasoanal\;length\;(cm)}$$

**Determination of Plasma Insulin Level:** was performed according to Flier et al. [18], using ELISA kits (DRG instruments).

Homeostasis Model Assessment of insulin resistance (HOMA-IR) was calculated by the following equation according to **Salgado et al.** [19]:

$$\text{HOMA-IR}=\text{Fasting}\;\text{Plasma}\;\text{Insulin}\;(\mu \text{U}/\text{mL})\;\text{x}\;\text{Fasting}\;\text{Plasma}\;\text{Glucose}\;\left(\text{mmol}/\text{L}\right)\;/22.5$$

#### Determination of Tissue Malondialdehyde (MDA) level

Small intestinal tissue homogenization was performed according to **Eissa et al.** [20], using the homogenizer: Karl Kolb, scientific technical supplies D−6072, Dreieich, West Germany. Tissue Malondialdehyde level was determined as an indicator of lipid peroxidation products in tissues, according to the technique of **Esterbauer and Cheeseman** [21].

**Determination of Catalase Activity in Small Intestinal Tissue:** was performed according to **Aebi** [22], by using calorimetric kits (Biodiagnostic, Egypt).

**Determination of tissue Glutathione Peroxidase (GPx) activity** was performed according to **Paglia and Valentine** [23], by using calorimetric kits (Biodiagnostic, Egypt).

### Histological examination

Dissected pieces from different small intestinal segments were fixed in 10% formalin for at least one week and were subjected to dehydration in ascending grades of alcohol, cleared in Xylol, impregnated in pure soft paraffin, and embedded in hard paraffin. Longitudinal sections of 7 μm thickness were cut by microtome and stained by Hematoxylin and Eosin [24], to study the general histological features.

## Statistical analysis

All results in the present study were expressed as mean ± SE of the mean. Statistical Package for the Social Sciences (SPSS, Inc., Chicago, IL, USA) program, version 20.0 was used. Differences were considered significant when P ≤ 0.05.

## Results

### Duodenal motility changes

**Fig 1:**
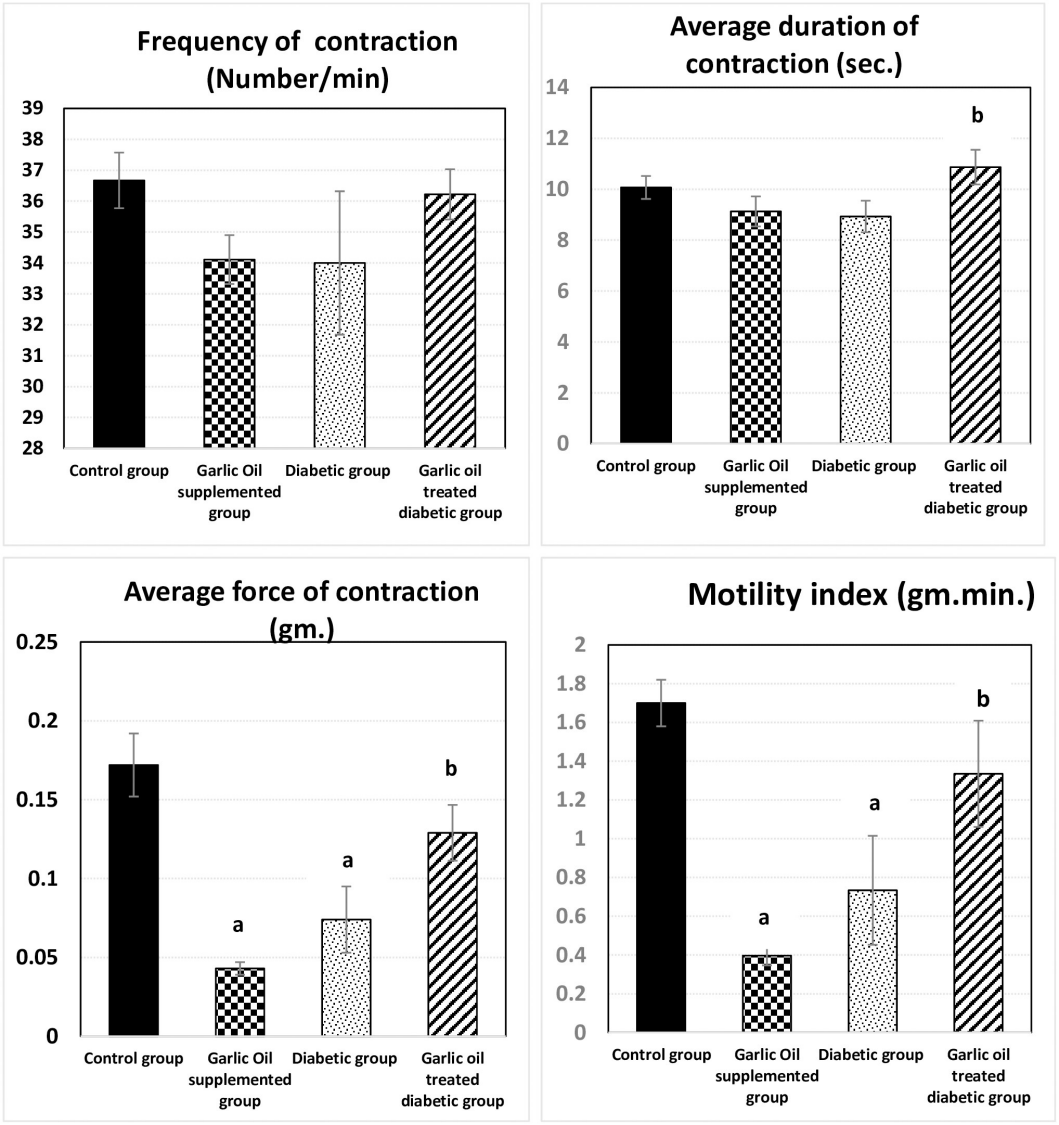
Changes in duodenal motility parameters [frequency of contraction (number of contractions / min.), average duration of contraction (sec.), average force of contraction (gm.), and motility index (gm. min.)] in the different studied groups. a: significance by LSD < 0.05 from the control group. b: significance by LSD < 0.05 from the diabetic group.

**Fig 2:**
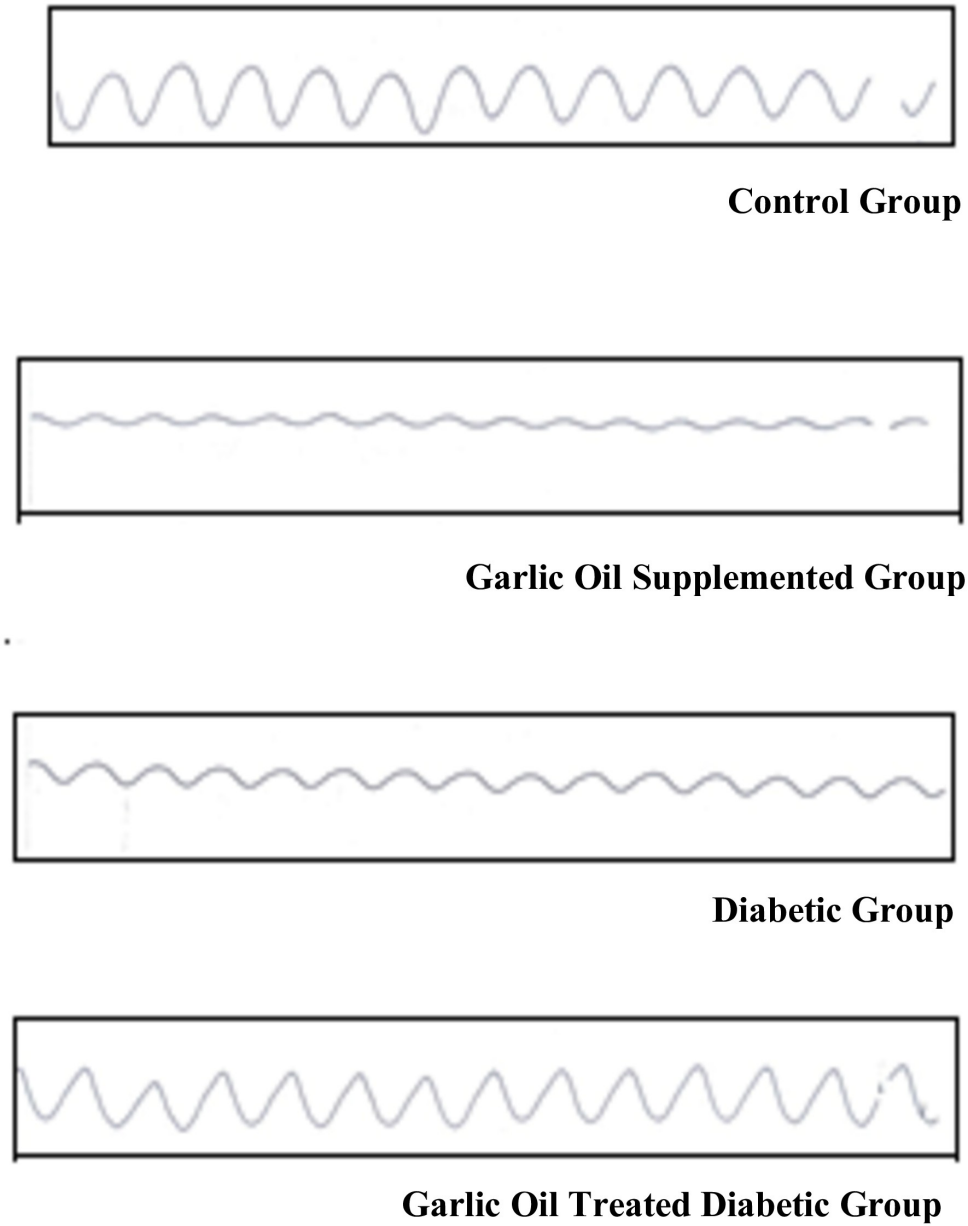
Traces of duodenal motility changes in the different studied groups.

As shown in Figs 1 and 2, the duodenal frequency of contractions and the average duration of contraction were insignificantly changed in all garlic oil-supplemented non-diabetic group (GO), diabetic group (D), and garlic oil-treated diabetic group (DG) when each was compared to the control group (C). However, the average duration of contraction was significantly increased in the DG group compared to the D group.

Both the average force of contraction and motility index were significantly lowered in the GO and D groups compared to the C group. However, they were significantly elevated in the DG group compared to the D group, to become insignificantly different from the C group.

### Jejunal motility changes

**Fig 3:**
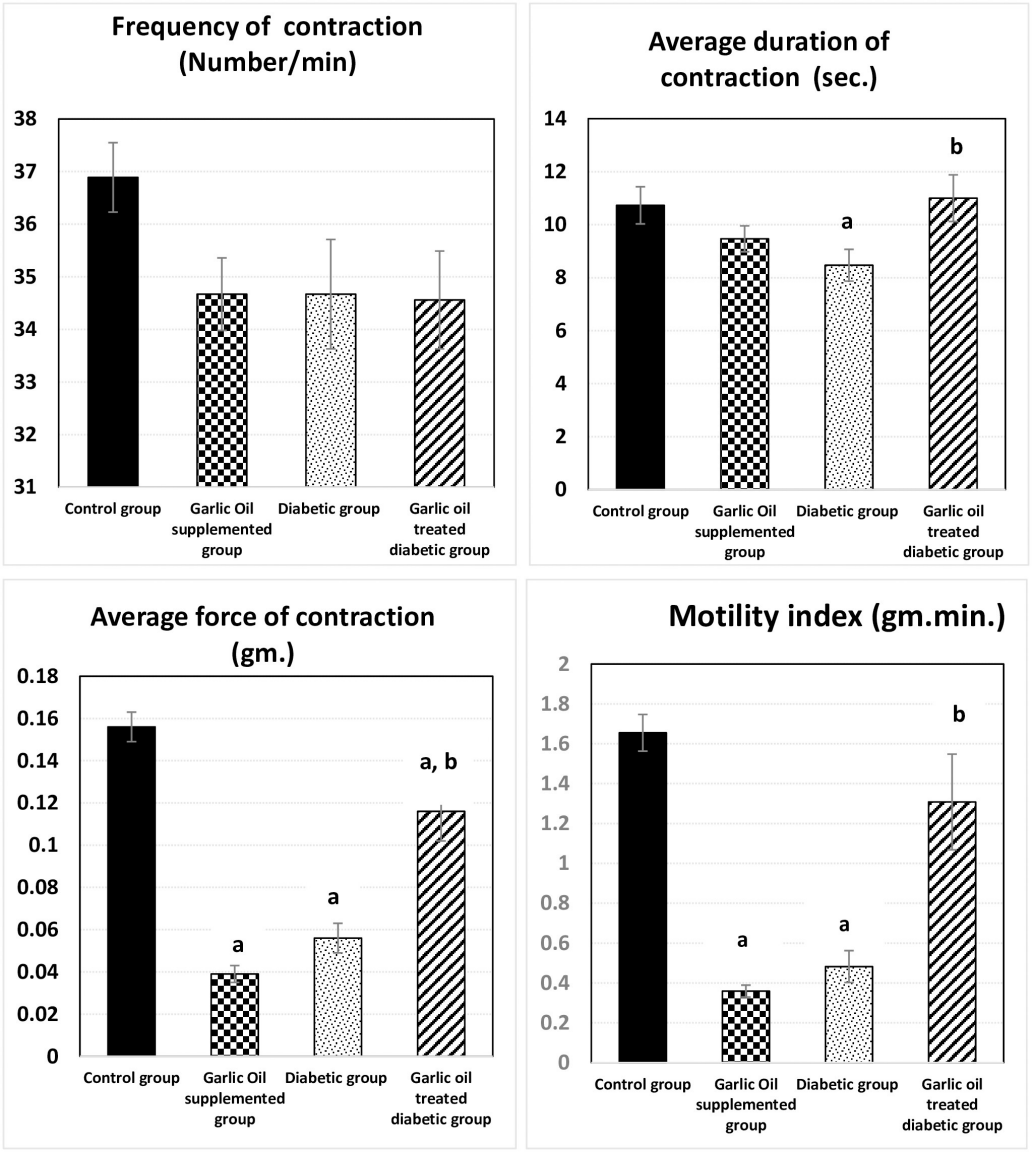
Changes in jejunal motility parameters [frequency of contraction (number of contractions / min.), average duration of contraction (sec.), average force of contraction (gm.) and motility index (gm. min.)] in the different studied groups. a: significance by LSD < 0.05 from the control group. b: significance by LSD < 0.05 from the diabetic group.

**Fig 4:**
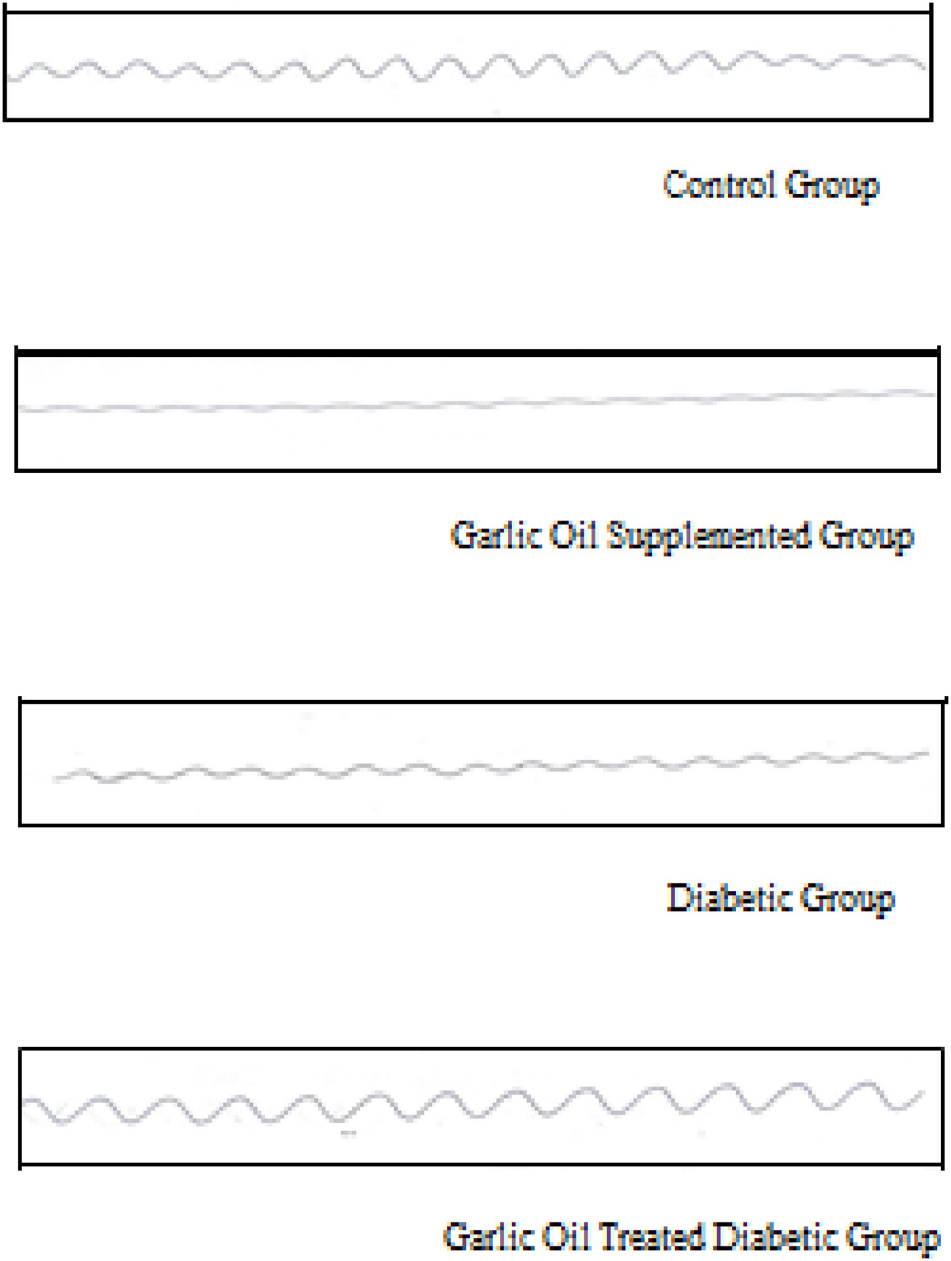
Traces of jejunal motility changes in the different studied groups.

As shown in Figs 3 and 4, non-significant changes were observed in the jejunal frequency of contraction when comparing all test groups to the control one. Similarly, insignificant changes in jejunal frequency of contraction were found between the DG and D groups. Also, the jejunal average duration of contraction was insignificantly changed in the GO and the DG groups compared to the C group. However, it was significantly reduced in the D group compared to the C group. Meanwhile, the jejunal average duration of contraction was significantly increased in the DG group compared to the D group.

The jejunal average force of contraction and motility index were significantly lowered in each of the GO group and the D group compared to the C group. Moreover, the DG group showed a significant decrease only in the average force of contraction compared to the C group. On the other hand, both the jejunal average force of contraction and the motility index were significantly elevated in the DG group compared to the D group.

### Ileal motility changes

**Fig 5:**
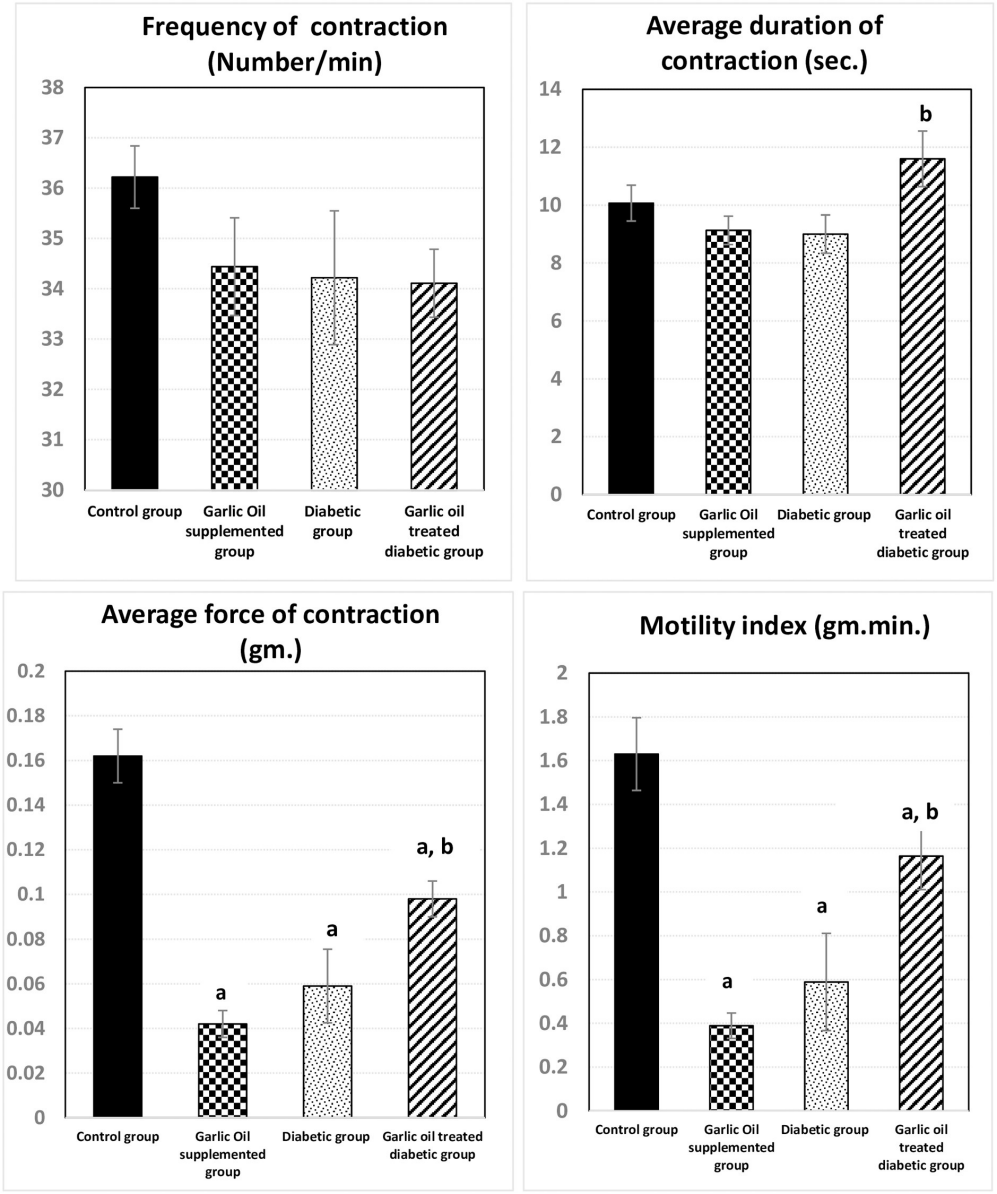
Changes in ileal motility parameters [frequency of contraction (number of contractions / min.), average duration of contraction (sec.), average force of contraction (gm.) and motility index (gm. min.)] in the different studied groups. a: significance by LSD < 0.05 from the control group. b: significance by LSD < 0.05 from the diabetic group.

**Fig 6:**
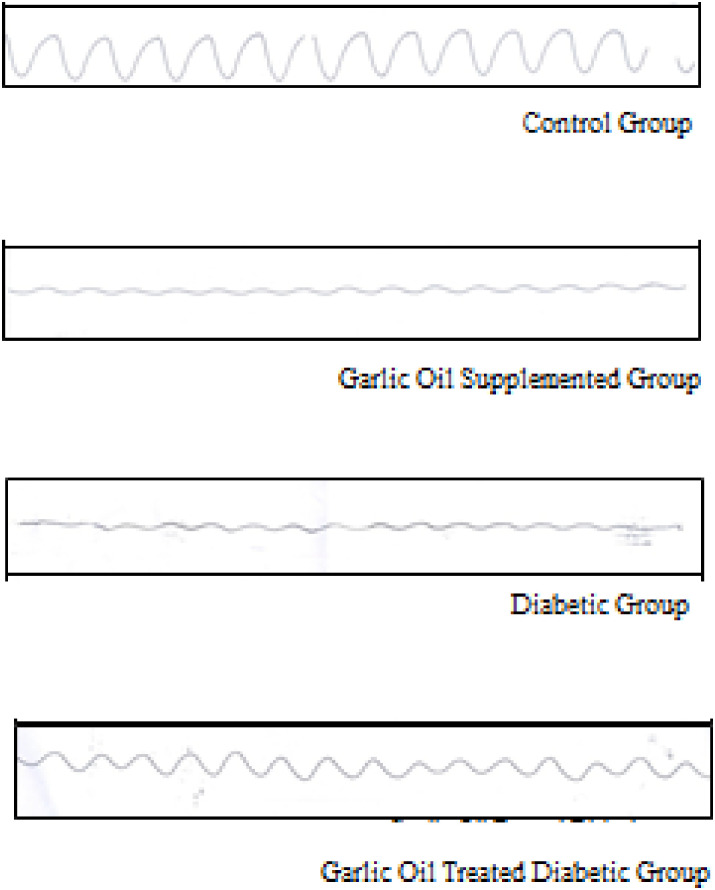
Traces of Ileal motility changes in the different studied groups.

As shown in Figs 5 and 6, the ileal frequency of contraction and the average duration of contraction showed non-significant changes in the GO, D, and DG groups compared to the C group. However, the ileal average duration of contraction was significantly elevated in the DG compared to the D group, without significant changes in the ileal frequency of contraction.

Both of ileal average force of contraction and motility index were significantly lowered in the GO, D, and DG groups compared to the C group. However, they were significantly elevated in the DG group compared to the D group.

### Changes in fasting blood glucose (FBG) and plasma insulin level, HOMA-IR and HbA1c

**Fig 7:**
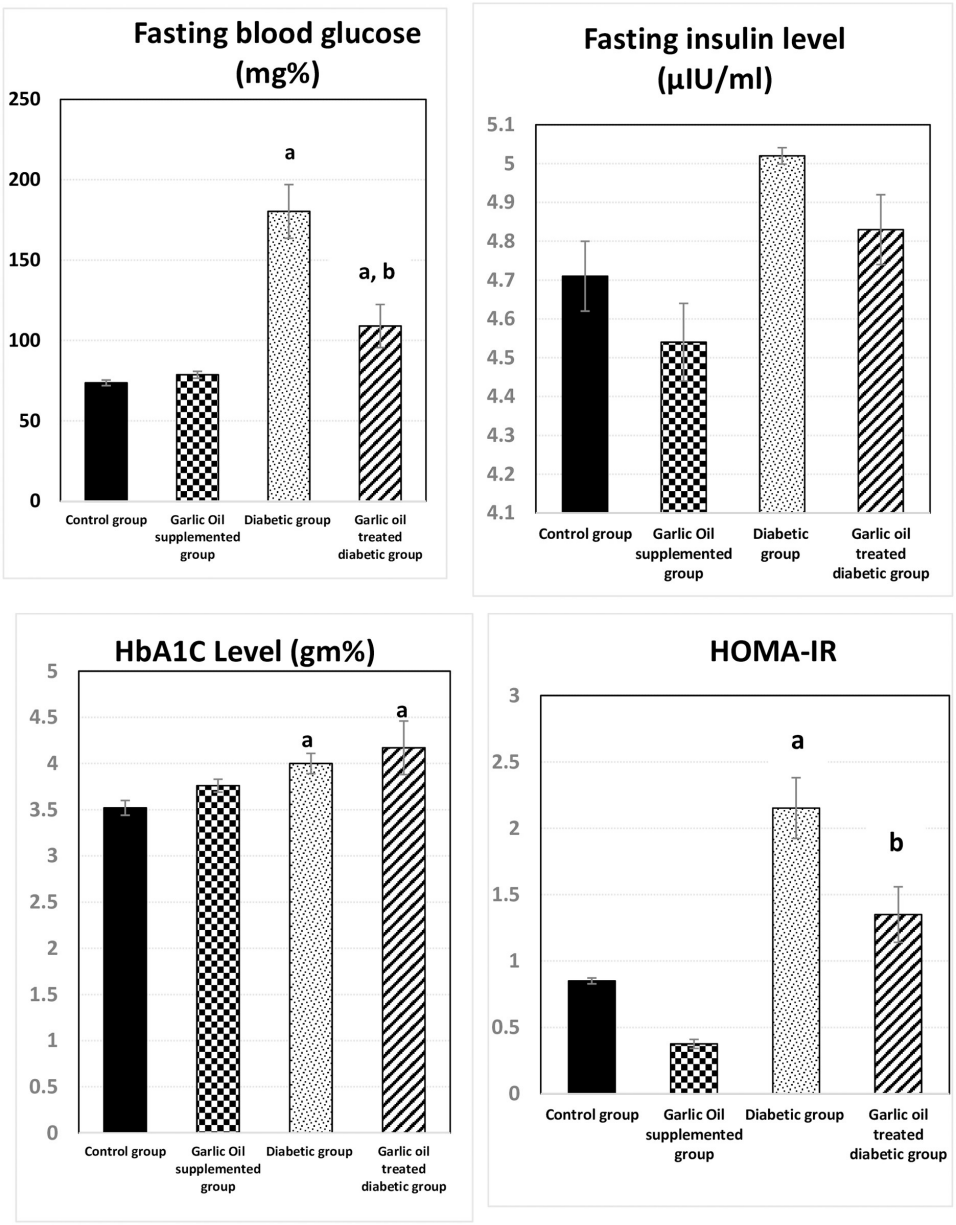
Changes in fasting blood glucose (mg%), fasting insulin level (μIU/ml), HOMA- IR, and HbA1c (gm%) in the different studied groups. a: significance by LSD < 0.05 from the control group. b: significance by LSD < 0.05 from the diabetic group.

As shown in Fig 7, FBG was significantly higher in the D and DG groups compared to the C group. On the other hand, it showed non-significant changes when comparing the GO group and the C group. However, it was significantly lower in the DG group than D group.

The fasting insulin level showed non-significant changes in the GO, D, and DG groups when compared to the C group, and in the GO, group compared to the D group. HOMA-IR score showed a significant rise in the D group compared to the C group. However, the GO and DG groups showed insignificant changes in the HOMA-IR score compared to the C group. Meanwhile, HOMA-IR score was significantly reduced in the DG group compared to the D group.

Although HbA1c was insignificantly changed in the GO group compared to the C group, it was significantly elevated in the D and DG groups compared to the C group. Non-significant changes in HbA1c were found in the DG group compared to the D group.

### Anthropometry measures

**Fig 8:**
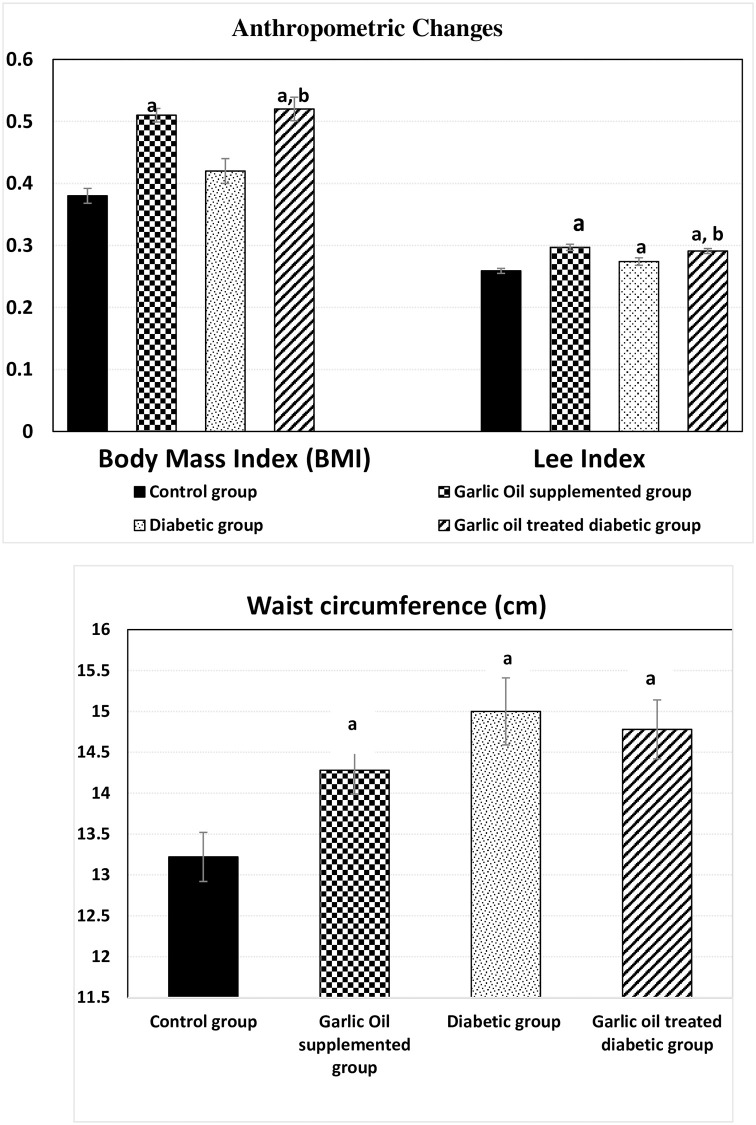
Changes in body mass index (BMI), Lee index and waist circumference in the different studied groups. a: significance by LSD < 0.05 from the control group. b: significance by LSD < 0.05 from the diabetic group.

Compared to the C group, body mass index (BMI) was significantly higher in the GO and DG groups, while it was insignificantly changed in the D group compared to the C group. Meanwhile, it was significantly elevated in the DG group compared to the D group, as shown in Fig 8.

Lee index was significantly higher in each of the GO, the D, and the DG groups compared to the C group. Similarly, it was significantly higher in the DG group compared to the D group.

Waist circumference was significantly higher in the GO, the D, and the DG groups when compared to the C group, although it was insignificantly changed in the DG group compared to the D group.

### Changes in markers of oxidative stress

#### Changes in Small Intestinal Malondialdehyde (MDA) level

**Fig 9:**
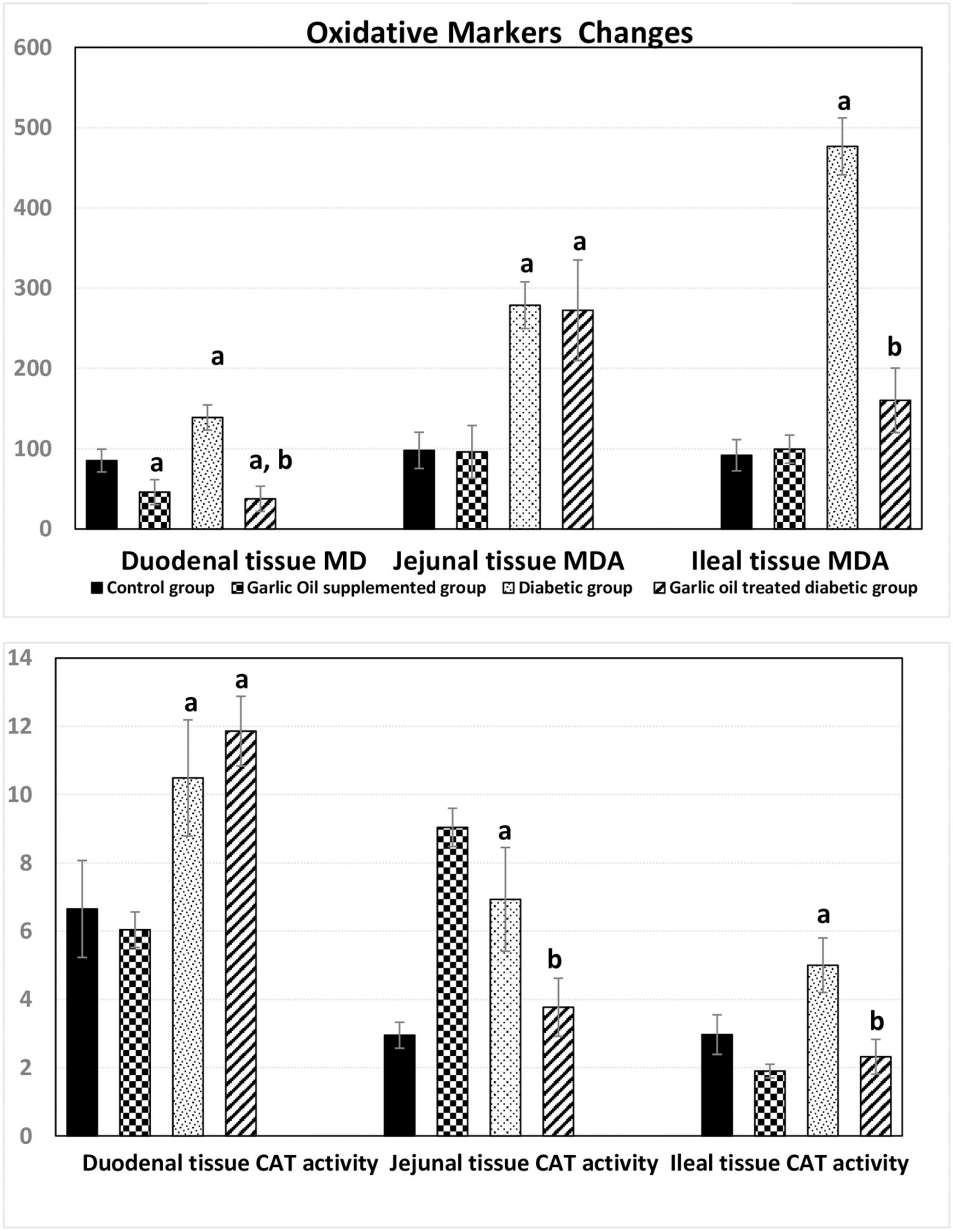
Changes in oxidative stress markers [Malondialdehyde level (MDA, nmol/gm wet tissue) and in catalase (CAT, U/gm wet tissue) activity] in the duodenum, jejunum and ileum in the different studied groups. a: significance by LSD < 0.05 from the control group. b: significance by LSD < 0.05 from the diabetic group.

As shown in Fig 9, the D group showed a significant rise in MDA levels in the duodenum, jejunum, and ileum when compared to the C group.

However, garlic oil supplementation had a significantly elevated in the duodenal tissue MDA compared to the controls despite non-significant changes in MDA levels in both jejunum and ileum.

On the other hand, garlic oil treatment in diabetic rats caused a significant reduction in MDA levels in both duodenum and jejunum compared to control ones; however, it did not reach the statistical level of significance in the ileum. Meanwhile, compared to the diabetic group, garlic oil treatment caused a significant reduction in MDA levels in the duodenum and ileum only.

#### Changes in Small Intestinal catalase (CAT) activity

As shown in Fig 9, garlic oil supplementation had insignificant changes in CAT activities in all small intestinal segments compared to the control rats. Meanwhile, the diabetic group showed significantly higher CAT activities in three regions of the small intestine compared to the control group.

However, garlic oil treatment in diabetic rats caused a significantly higher CAT activity only in the duodenum when compared to the controls. On comparing DG and D groups, CAT activities were significantly lowered in both jejunum and ileum, despite being insignificantly changed in the duodenum.

#### Changes in small intestinal glutathione peroxidase activity

As shown in Table 1, duodenal tissue glutathione peroxidase was insignificantly changed in each of the garlic oil-treated groups, the diabetic group, and the garlic oil-treated diabetic group compared to the control group. However, duodenal tissue glutathione peroxidase was significantly decreased in the garlic oil-treated diabetic group compared to the diabetic group.

**Table 1:** Mean ± SEM of small intestinal glutathione peroxidase activity, GSH-PX (U/gm wet tissue) in the different studied groups.

Group	Control group	Garlic Oil-supplemented group	Diabetic group	Garlic Oil-treated Diabetic group
**Duodenal tissue GSH-PX (U/gm wet tissue)** **P** P*	950.49 ±266.72	953.96 ±104.34 NS	1471.8 ±374.58 NS	550.18 ±141.46 NS <0.02
**Jejunal tissue GSH-PX (U/gm wet tissue)** **P** P*	1924.6 ±561.04	680.89 ±147.83 <0.02	1771.2 ±313.84 NS	550.47 ±113.88 <0.01 <0.02
**Ileal tissue GSH-PX** **(U/gm wet tissue)** **P** P*	878.01 ±189.27	1015.8 ±129.65 NS	1629.4 ±406.58 <0.05	852.72 ±161.17 NS <0.05

Values are expressed as means ±SEM

P: Significance by LSD at P ≤0.05 from the control group

P*: Significance by LSD at P **<**0.05 from the diabetic group

NS: Not significant

Jejunal tissue glutathione peroxidase was insignificantly changed in the diabetic group while it was significantly decreased in each of the garlic oil-supplemented group and the garlic oil-treated diabetic group when each group was compared to the control group. Moreover, jejunal tissue glutathione peroxidase was significantly decreased in the garlic oil-treated diabetic group compared to the diabetic group.

Ileal tissue glutathione peroxidase was significantly elevated in the diabetic group compared to the control group, but non-significant changes in ileal glutathione peroxidase were detected in the garlic oil-supplemented group and the garlic oil-treated diabetic group compared to the control group. Meanwhile, ileal tissue glutathione peroxidase was significantly reduced in the garlic oil-treated diabetic group compared to the diabetic group.

### Histological findings

#### Duodenal changes

**Fig 10:**
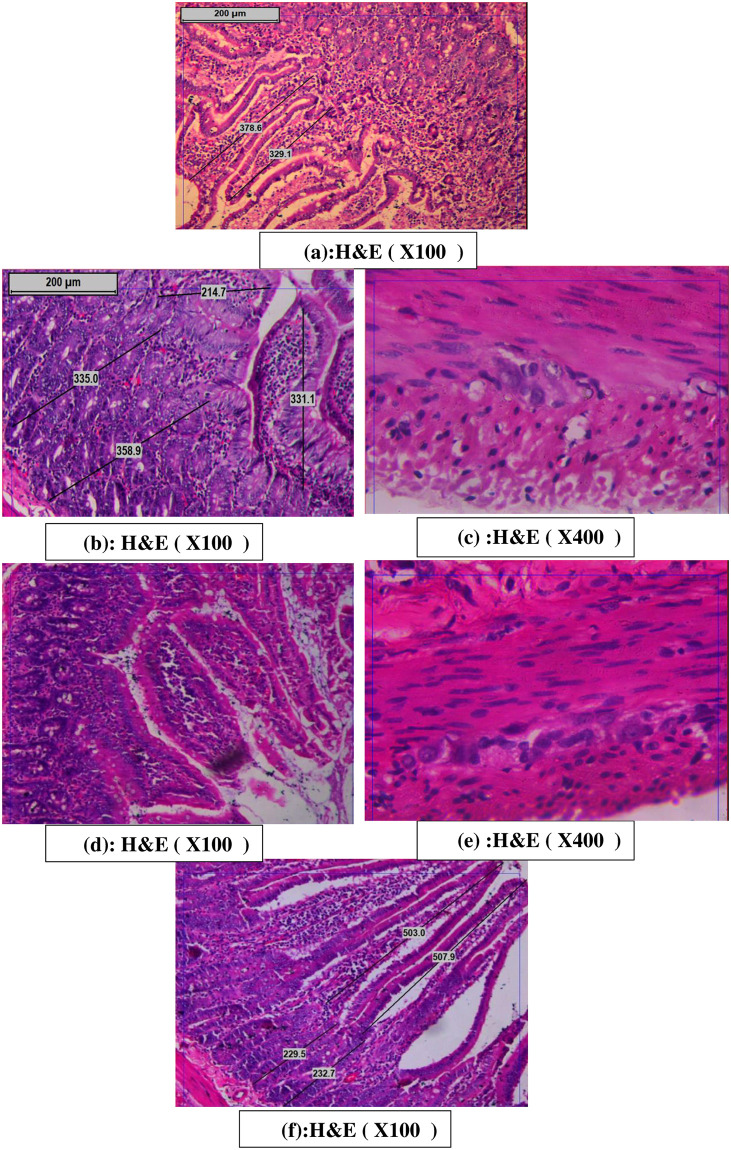
Photomicrographs of histological changes in the duodenum in the different studied groups; the control group showed regular villi and crypts with basophilic cytoplasm and vesicular nuclei (fig a). The diabetic group showed markedly irregular villi, and mononuclear cell infiltration, some of the cells covering the villi appear deeply stained with pyknotic nuclei (fig b), also, its myenteric nervous plexus showed vacuolated cytoplasm with pyknotic nuclei (fig c). The garlic oil-treated diabetic group showed a prominent decrease in mononuclear cell infiltration (fig d), and its myenteric nervous plexus showed basophilic cytoplasm with normal vesicular nuclei (fig e). Garlic the oil-supplemented group showed regular villi and crypts with basophilic cytoplasm and vesicular nuclei (fig f).

As shown in Fig 10, microscopic examination of H&E-stained duodenum of the control group showed regular villi and crypts with basophilic cytoplasm and vesicular nuclei (Fig 10a and 10b). The diabetic group showed markedly irregular villi, and mononuclear cell infiltration, some of the cells covering villi appear deeply stained with pyknotic nuclei (Fig 10c). Also, the duodenal myenteric nervous plexus in the diabetic group showed vacuolated cytoplasm with pyknotic nuclei (Fig 10d). The garlic oil-treated diabetic group showed a prominent decrease in mononuclear cell infiltration (Fig 10e), and its myenteric nervous plexus showed basophilic cytoplasm with normal vesicular nuclei (Fig 10f). The garlic oil-supplemented group showed regular villi and crypts with basophilic cytoplasm and vesicular nuclei. Myenteric nervous plexus showed normal vesicular nuclei (Fig 10g).

#### Jejunal changes

**Fig 11:**
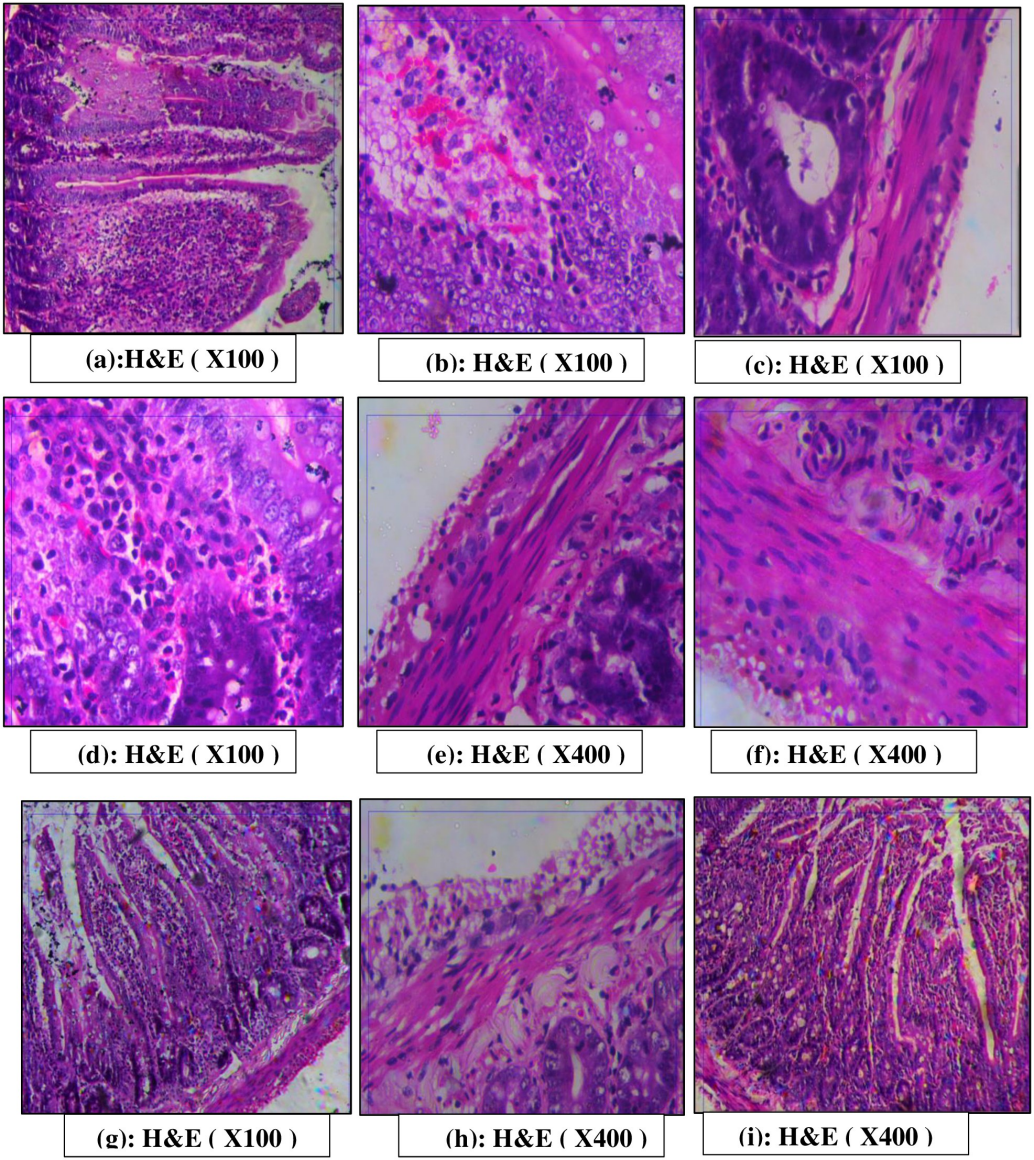
Photomicrographs of histological changes in the jejunum in the different studied groups. The control group showed vesicular nuclei with basophilic cytoplasm. The diabetic group had villi and crypts with lost nuclei, hyperplasia, and mononuclear cell infiltration (fig a), areas of hemorrhage and areas of congestion (fig b), apoptotic cells, and mitotic figures (fig c). Many neutrophils, lymphocytes, and macrophages were observed in the diabetic group (fig d), also, its myenteric nervous plexus showed scarce nerve cells, not in groups, of small size with deep shrunken nuclei (fig e). The garlic oil-treated diabetic group showed regular villi and crypts with basophilic cytoplasm and vesicular nuclei (fig f) with a prominent decrease in mononuclear cell infiltration, and very minimal necrotic areas (fig g), also its myenteric nervous plexus showed decreased vacuolated cells with increased eccentric nuclei (fig h). The garlic oil-supplemented group showed regular villi and crypts with basophilic cytoplasm and vesicular nuclei, its myenteric nervous normal vesicular nuclei (fig i).

As shown in Fig 11, the control group showed vesicular nuclei with basophilic cytoplasm. The diabetic group had villi and crypts with lost nuclei, hyperplasia, and mononuclear cell infiltration (Fig 11a), areas of hemorrhage and areas of congestion (Fig 11b), apoptotic cells, and mitotic figures (Fig 11c). Many neutrophils, lymphocytes, and macrophages were observed in the diabetic group (Fig 11d), additionally, its myenteric nervous plexus showed scarce nerve cells, not in groups, of small size with deep shrunken nuclei (Fig 11e). The garlic oil-treated diabetic group showed regular villi and crypts with basophilic cytoplasm and vesicular nuclei (Fig 11f) with a prominent decrease in mononuclear cell infiltration, very minimal necrotic areas (Fig 11g), also its myenteric nervous plexus showed decreased vacuolated cells with increased eccentric nuclei (Fig 11h). The garlic oil-supplemented group showed regular villi and crypts with basophilic cytoplasm and vesicular nuclei, its myenteric nervous normal vesicular nuclei (Fig 11i).

#### Ileal changes

**Fig 12:**
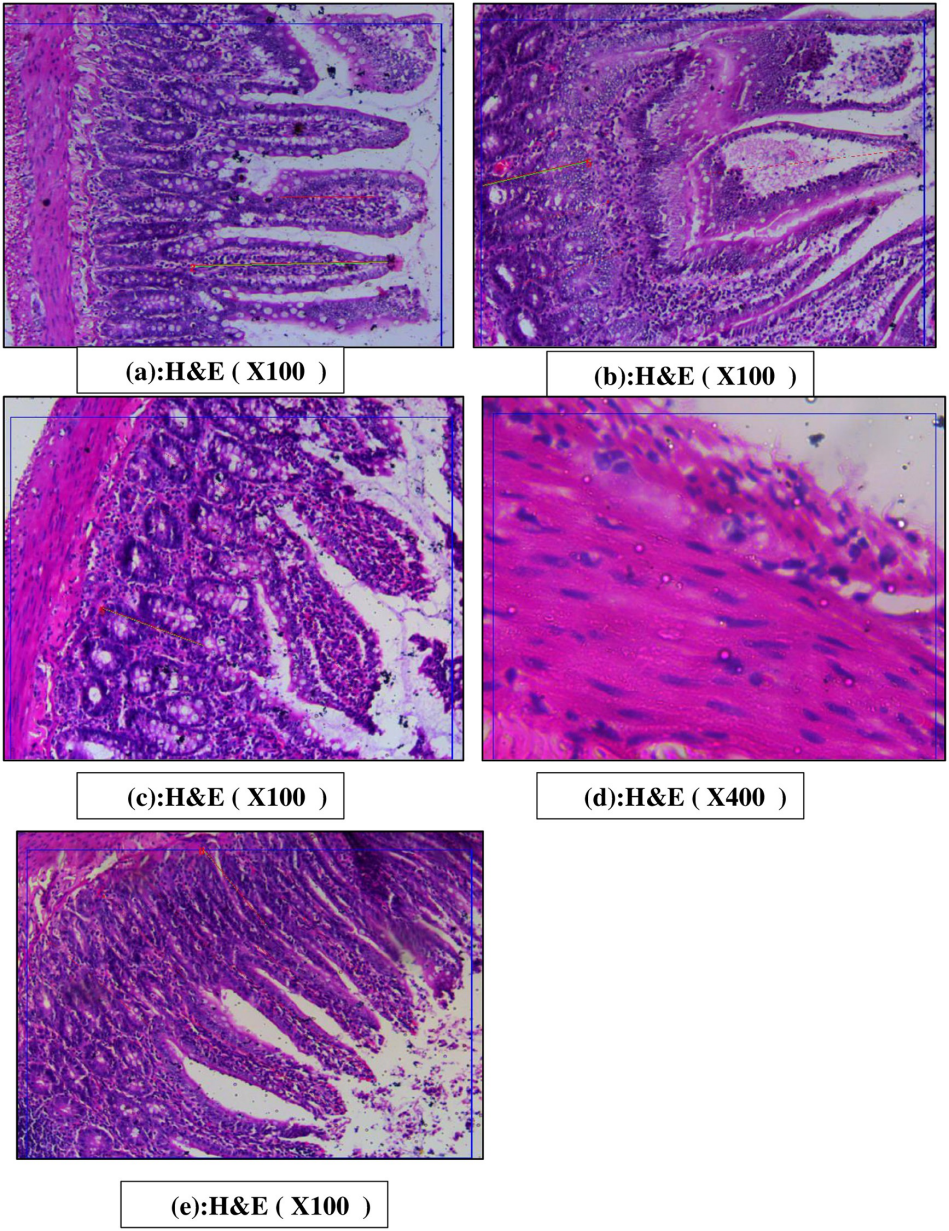
Photomicrographs of histological changes in the ileum in the different studied groups. The control group showed regular villi and crypts with basophilic cytoplasm and vesicular nuclei (fig a), and its myenteric nervous plexus showed normal vesicular nuclei. The diabetic group had widened distorted villi with necrotic tissue in the center, and dense mononuclear cell infiltration (fig b), its myenteric nervous plexus shows scarce nerve cells not in groups; they are small with deep shrunken nuclei. Also, there are marked distortions in the muscle layer. The garlic oil-treated diabetic group showed villi that regained their shape, and muscles retained their normal arrangement; however, there was still mononuclear cell infiltration (fig c). Its myenteric nervous plexus showed basophilic cytoplasm with normal vesicular nuclei (fig d). The garlic oil-supplemented group showed normal villi and crypts, but there is mononuclear cell infiltration (fig e). The myenteric nervous plexus showed normal vesicular nuclei.

As shown in Fig 12, the control group showed regular villi and crypts with basophilic cytoplasm and vesicular nuclei (Fig 12a), and its myenteric nervous plexus showed normal vesicular nuclei. The diabetic group had widened distorted villi with necrotic tissue in the center, and dense mononuclear cell infiltration (Fig 12b), its myenteric nervous plexus showed scarce nerve cells not in groups; they were small with deep shrunken nuclei. Also, there were marked distortions in the muscle layer. The garlic oil diabetic treated group showed villi that regained their shape, and muscles retained their normal arrangement, however, there was still mononuclear cell infiltration (Fig 12c), and its myenteric nervous plexus showed basophilic cytoplasm with normal vesicular nuclei (Fig 12d). Garlic oil supplemented group showed normal villi and crypts but there was mononuclear cell infiltration (Fig 12e), its myenteric nervous plexus showed normal vesicular nuclei.

## Discussion

In the present study, impaired small intestinal motility was observed in the diabetic group. This decline in the force of contraction of various small intestinal segments could potentially be attributed to the direct impact of free radicals and oxidative stress on the smooth muscles of the small intestine, as indicated by higher levels of malondialdehyde (MDA) in the duodenal, jejunal, and ileal regions. These findings are consistent with those of **Hernandez et al.** [25], who proposed that hydrogen peroxide induces relaxation of smooth muscle by hyperpolarization through the activation of large-conductance calcium-activated potassium channels.

Likewise, the motility indices of the duodenum, jejunum, and ileum were significantly reduced in the diabetic group, in line with the observations made by **Hu and Feng** [26], which could be attributed to a decrease in vagal cholinergic activity under hyperglycemic conditions.

Furthermore, the noteworthy decrease in the average force of contraction and motility index in the small intestine of diabetic rats could potentially be due to the involvement of myosin light chain kinase (MLCK). Altered levels of cytosolic calcium ions (Ca2+) can affect smooth muscle contractility through the phosphorylation of the myosin light chain, which is activated by MLCK. When the expression of MLCK is decreased in gastrointestinal tissues in the context of diabetes mellitus (DM), gastrointestinal dysfunction follows [26].

However, the changes in the motility index induced by diabetes in the current study contradict the findings of **Kim et al.** [8], who reported no effect of diabetes mellitus on the motility index.

Insignificant changes in the frequency of contractions in the studied different small intestinal segments align with the results reported by **Lammers et al.** [27]. They observed that slow wave frequencies, velocities, and amplitudes remained unchanged at all stages of diabetes, which were explained by an insufficient depletion of interstitial cells of Cajal to affect slow wave propagation. Additionally, it could be suggested that small intestinal dysfunction with unchanged slow wave frequencies may be due to impairment of the enteric nervous system or the muscle cells themselves.

The histopathological observations in the diabetic group can elucidate the decrease in the average force of contraction and motility index in all small intestinal segments. This notion is supported by **Locke** [28], who documented that gastrointestinal manifestations in diabetic patients can be attributed to disrupted motor function caused by irreversible autonomic neuropathy. Additionally, diabetic autonomic neuropathy, a primary contributor to digestive disorders in diabetes mellitus, can induce alterations in each segment of the gastrointestinal tract [29]. Furthermore, **Jones et al.** [30] reported that inadequate glycemic control may be a significant factor contributing to gastrointestinal dysfunction. In the present study, elevated levels of HbA1c in the diabetic group indicated poor glycemic control.

Furthermore, the presence of mononuclear infiltration was observed in the duodenal, jejunal, and ileal tissues of the diabetic group. **Demedts et al.** [31] suggested that diabetic rats experience altered intestinal motor control, which can be explained by the loss of nitrergic control and potentially associated with transient inflammatory infiltrates. The presence of pyknotic nuclei in the villi and myenteric plexus of all small intestinal segments in the diabetic group suggests the occurrence of apoptosis, as reported in the study conducted by **Anitha et al.** [32] on the myenteric plexus in streptozotocin-induced diabetic mice. Similarly, **He et al.** [33] found that diabetic mice induced by streptozotocin exhibited apoptosis of enteric neurons in the myenteric plexus as well as a depletion of interstitial cells of Cajal in the jejunum.

In the current study, fasting insulin levels showed insignificant changes in the diabetic group, which contradicts the findings of **Steinberger and Daniels** [34]. However, fasting blood glucose, HOMA-IR score, HbA1c, Lee index, and waist circumference were significantly elevated in the diabetic group, consistent with the results reported by **Kim et al.** [35]. Although BMI did not display significant changes in the diabetic group in the present study, DM has been reported to be associated with all levels of BMI according to **Bays et al.** [36].

Furthermore, in the present study, the average force of contraction of the studied segments was significantly reduced in the non-diabetic group supplemented with garlic oil, similar to the findings of **Aqel et al.** [37]. This reduction could be attributed to the inhibition of spontaneous intestinal movements by garlic oil, which may depend on extracellular calcium influx. **Aqel et al.** [37], also, demonstrated significant reductions in the motility index of the duodenum, jejunum, and ileum in rats supplemented with garlic oil. Moreover, **Ferri et al.** [38] found that synthetic ajoene, an organosulfur compound derived from garlic, specifically blocked the cell cycle and subsequently impaired smooth muscle cell functions, thereby affecting smooth muscle proliferation.

Notably, the administration of garlic oil to diabetic rats resulted in an improvement in the average duration of contraction, average force of contraction, and motility index in all segments of the small intestine. This improvement can be attributed to the antioxidant and hypoglycemic effects of garlic oil in diabetic rats. **Chen et al.** [39] similarly suggested that extracts of black garlic stimulate gastrointestinal peristalsis by increasing the levels of serotonin.

Furthermore, histological examination revealed that the duodenum, jejunum, and ileum of the diabetic group treated with garlic oil exhibited restored villi and muscles with a normal arrangement. Additionally, there was a decrease in mononuclear cell infiltration and the myenteric nervous plexuses displayed basophilic cytoplasm with normal vesicular nuclei. These improvements in histopathological changes observed in diabetic rats treated with garlic oil can be mediated by its antihyperglycemic and antioxidant effects, as demonstrated in the current study.

The antidiabetic effects of garlic oil are consistent with the findings of **Liu et al.** [40]. Both garlic oil and diallyl trisulfide have been shown to improve glycemic control in diabetic rats by increasing insulin secretion and enhancing insulin sensitivity [41]. Garlic may enhance glycemic control, particularly at higher doses, by increasing pancreatic insulin secretion from β cells or facilitating the release of bound insulin, thereby acting as an insulin secretagogue in diabetic rats [42]. Additionally, garlic has been found to effectively reduce blood glucose levels in streptozocin-induced diabetic rats [43]. Allicin, a sulfur-containing amino acid present in garlic, may also alleviate diabetic conditions in rats, exhibiting effects similar to those of glibenclamide and insulin [44]. The antidiabetic effects of garlic oil may also involve the improvement of GLUT4 expression and the reversal of insulin resistance [40].

However, the observed glucose-lowering effect of garlic oil supplementation in the present study contradicts the findings of [45], who reported no change in blood glucose levels following garlic oil administration in diabetes. This discrepancy may explain the insignificant changes observed in fasting insulin, fasting blood glucose, HOMA-IR, and HbA1c in the non-diabetic group supplemented with garlic oil compared to the control group. Nevertheless, **Anwar and Meki** [46] reported that treatment of diabetic rats with garlic oil led to a partial decrease in plasma glucose levels, although not reaching control levels.

The persistently higher levels of HbA1c in the treated diabetic rats observed in this study contradict the hypoglycemic effects of garlic oil on HbA1c levels reported by **Phil et al.** [47]. The increased levels of glycosylated hemoglobin observed in diabetic patients, which are directly proportional to blood glucose levels [48], suggest increased oxidative stress due to hyperglycemia.

The treatment of diabetes with garlic oil in this study improved insulin resistance, possibly through the action of diallyl disulfide (DADS) present in garlic [49], which promoted increased insulin secretion and improved insulin sensitivity [41]. The reduced HOMA-IR and MDA levels observed in the diabetic rats treated with garlic oil can be explained by the findings of **Liu et al.** [50], who attributed these effects to the downregulation of oxidative stress and the synthesis of proinflammatory cytokines induced by Allium Sativum supplementation, which relates to its overall enhancement of the innate immune response.

The non-diabetic group supplemented with garlic oil and the diabetic group treated with garlic oil demonstrated a significant increase in BMI, Lee Index, and waist circumference. These findings differ from the study of **Wu et al.** [51] and of **Superko and Krauss** [52]. The variation could be attributed to the use of different rat strains and experimental models for inducing diabetes mellitus.

In this study, noticeable oxidative stress was observed in all segments of the small intestine in the diabetic group, which is consistent with the findings of **Moussa** [9] and **Tiwari et al.** [53]. This can be explained by the formation of free radicals in diabetes through glucose autoxidation and non-enzymatic glycation of proteins [54]. The abnormally high levels of free radicals and the simultaneous decline in antioxidant defence systems can lead to damage to cellular organelles and enzymes, increased lipid peroxidation, and the development of complications associated with diabetes mellitus. These effects may be mediated by the increased activity of the enzyme fatty acyl-coenzyme A oxidase, which initiates the β-oxidation of fatty acids and leads to lipid peroxidation.

The antioxidant effect of garlic oil in the treated diabetic group is consistent with the findings of **Hassan et al.** [10] and **Zhang et al.** [54]. This effect may be attributed to the scavenging of free radicals, leading to a decline in nitric oxide levels and lipid peroxidation. Additionally, **Anwar and Meki** [46] reported that garlic contains numerous sulfur-containing compounds that act as a powerful antioxidant system and minimize intracellular oxidative damage. However, the MDA levels in the jejunum showed insignificant changes in the garlic oil-treated diabetic group compared to the diabetic ones. This could be due to different responses of small intestinal tissues to oxidative stress.

Furthermore, catalase (CAT) activities in all segments of the small intestine were significantly increased in the diabetic group, similar to the study conducted by **Kakkar et al.** [55], who attributed the increased CAT activity in the liver and pancreas to prominent oxidative stress. Similarly, garlic oil supplementation restored CAT activity levels in oxidative status in male albino rats [10].

In the garlic oil-supplemented group, MDA levels were significantly decreased in the duodenum compared to the controls, while there were no significant changes in the activities of CAT in the three regions of the small intestine. Additionally, there were significant reductions in CAT activities only in the jejunum and ileum of garlic oil-treated diabetic rats compared to untreated diabetic rats. These findings disagree with those of **Zeng et al.** [56], who found an increase in CAT activity following treatment with Diallyl trisulfide (DATS). This discrepancy may be due to the short duration of garlic oil treatment.

The activity of glutathione peroxidase (GSH-Px) in the duodenum and jejunum showed insignificant changes in the diabetic group, which agrees with the findings of **Moussa** [9]. However, GSH-Px activity was significantly elevated in the ileum of the diabetic group, contradicting the findings of **Tiwari et al.** [53]. They suggested that this abnormal GSH status is involved in β-cell dysfunction and the pathogenesis of long-term complications of diabetes.

The decrease in GSH-Px activity in the garlic oil-treated diabetic group is consistent with the findings of **Chen et al.** [57], who suggested that garlic oil has a dose-dependent decline in GSH-Px activity. The variation between these studies may be due to differences in the target organs studied and the exposure of animals to toxins. Although GSH-Px activity was decreased by garlic oil, it was suggested that the concomitant increase in glutathione-S transferase activity, which is a selenium-independent GSH-Px, could compensate partly for the loss of selenium-dependent GSH-Px by reducing various organic hydroperoxides to alcohols.

It is important to note that autonomic neuropathy has been suggested to affect small intestinal function in diabetes [58]. **Zanoni et al.** [6] reported that elevated sorbitol production in diabetes mellitus can alter intracellular osmolality, leading to neuronal death. This could potentially be one of the underlying mechanisms for the motility disorders observed in this study, although it was not specifically investigated. Moreover, diabetes-induced mucosal disruption was observed, which could also be related to autonomic neuropathy. Reduced mesenteric perfusion, as seen in the diabetic group in the duodenum, jejunum, and ileum, could contribute to the reduction in villi, as reported by **De Las Casas and Finley** [59].

The link between autonomic neuropathy and oxidative stress in diabetes mellitus is further supported by the work of Kunze **and Furness** [60], who reported that the intrinsic neurons of the intestine, which control secretion and motility in the muscle, could be potential targets of reactive oxygen species (ROS). Therefore, the observed oxidative stress in diabetes mellitus, as evidenced by elevated MDA levels in the small intestinal segments, could be alleviated by garlic oil treatment. Also, autonomic neuropathy was observed, in the current study, in the diabetic group by myenteric plexus damage, which was also restored by garlic oil treatment. In addition, it was found that increased production of sorbitol in hyperglycemic conditions could result in edema and rupture of the nerve sheath, thereby reducing nerve impulse conduction [7]. In diabetes mellitus, advanced glycosylation end products are produced, resulting in morphological changes that occur during hyperglycemia, leading to gastrointestinal tract remodelling [61]. The aforementioned ameliorating effects of garlic oil could be suggested to mitigate the harmful oxidative effects of diabetes mellitus through its antioxidant, anti-inflammatory and, antiapoptotic effects [62–64].

## Conclusion

Diabetes mellitus resulted in a reduction in the force of contraction and motility index in the small intestine in rats, which may be caused by autonomic neuropathy and/or oxidative stress. Such changes were alleviated by garlic oil treatment.

### Recommendations

Garlic and its derivatives can be further studied in diabetes-induced digestive alterations as much human research has demonstrated the beneficial effects of garlic in diabetes mellitus-associated derangements such as endothelial dysfunction, vascular inflammation, oxidative stress, dyslipidaemia, and insulin resistance in diabetic persons [65–72]. Future research could further investigate the specific molecular mechanisms through which garlic oil positively affects the pathogenesis of Type II diabetes.

## Supporting information

S1 File Tables 2–29 are individual values of each group in all studied parameters in this study. (DOCX)

## Abbreviations

AS: Allium Sativum
CAT: Catalase
DADS: Diallyl disulphide
DM: Diabetes mellitus
FBG: Fasting blood glucose
GSH-Px: Glutathione peroxidase
HOMA-IR: Homeostasis model assessment of insulin resistance
i.p: intraperitoneal
MDA: Malondialdehyde
ROS: reactive oxygen species
SOD: Superoxide dismutase
STZ: Streptozotocin
