# Supplementary material for: Garlic oil improves small intestinal motility in experimentally induced type II diabetes mellitus in female Wistar rats
Source: PLoS One. 2024 Apr 17;19(4):e0301621. doi: 10.1371/journal.pone.0301621 (PMC11023395; doi:10.1371/journal.pone.0301621)
Supplement: S1 File — (DOCX) [file pone.0301621.s001.docx]

**Table (2):** Duodenal motility parameters [frequency of contraction (no. of contractions / min.), average duration of contraction (sec.), average force of contraction (gm.) and motility index (gm.min.)] in control group.

| **Motility index (gm.min.)** | **Average force of contraction**  **(gm.)** | **Average duration of contraction (sec.)** | **Frequency of contraction (/min)** | **Rat**  **No.** |
| --- | --- | --- | --- | --- |
| 2.34 | 0.26 | 9 | 40 | 1 |
| 2.122 | 0.221 | 9.6 | 38 | 2 |
| 1.966 | 0.182 | 10.8 | 40 | 3 |
| 1.42 | 0.182 | 7.8 | 34 | 4 |
| 1.521 | 0.169 | 9 | 36 | 5 |
| 1.326 | 0.13 | 10.2 | 38 | 6 |
| 1.404 | 0.13 | 10.8 | 35 | 7 |
| 1.482 | 0.13 | 11.4 | 32 | 8 |
| 1.716 | 0.143 | 12 | 37 | 9 |
| **1.7**  **±0.12** | **0.172**  **±0.02** | **10.07**  **±0.45** | **36.67**  **±0.9** | **Mean**  **±SEM** |

**Table (3):** Duodenal motility parameters [frequency of contraction (no. of contractions / min.), average duration of contraction (sec.), average force of contraction (gm.) and motility index (gm.min.)] in garlic supplemented group.

| **Motility index (gm.min.)** | **Average force of contraction**  **(gm.)** | **Average duration of contraction (sec.)** | **Frequency of contraction (/min)** | **Rat**  **No.** |
| --- | --- | --- | --- | --- |
| 0.624 | 0.065 | 9.6 | 34 | 1 |
| 0.499 | 0.052 | 9.6 | 32 | 2 |
| 0.562 | 0.052 | 10.8 | 33 | 3 |
| 0.203 | 0.026 | 7.8 | 36 | 4 |
| 0.374 | 0.039 | 9.6 | 31 | 5 |
| 0.312 | 0.052 | 6 | 39 | 6 |
| 0.468 | 0.039 | 12 | 35 | 7 |
| 0.304 | 0.039 | 7.8 | 34 | 8 |
| 0.234 | 0.026 | 9 | 33 | 9 |
| **0.397**  **±0.05**  **<0.001** | **0.043**  **±0.004**  **<0.001** | **9.13**  **±0.59**  **NS** | **34.11**  **±0.79**  **NS** | **Mean**  **±SEM**  **P** |

P: Significance by LSD at P< 0.05 from control group.

NS: Not significant.

**Table (4):** Duodenal motility parameters [frequency of contraction (no. of contractions / min.), average duration of contraction (sec.), average force of contraction (gm.) and motility index (gm.min.)] in diabetic group.

| **Motility index (gm.min.)** | **Average force of contraction**  **(gm.)** | **Average duration of contraction (sec.)** | **Frequency of contraction (/min)** | **Rat**  **No.** |
| --- | --- | --- | --- | --- |
| 0.328 | 0.039 | 8.4 | 42 | 1 |
| 0.257 | 0.039 | 6.6 | 33 | 2 |
| 0.374 | 0.052 | 7.2 | 37 | 3 |
| 0.562 | 0.052 | 10.8 | 34 | 4 |
| 2.948 | 0.234 | 12.6 | 17 | 5 |
| 0.608 | 0.078 | 7.8 | 38 | 6 |
| 0.499 | 0.052 | 9.6 | 34 | 7 |
| 0.328 | 0.039 | 8.4 | 34 | 8 |
| 0.702 | 0.078 | 9 | 37 | 9 |
| **0.734**  **±0.281**  **<0.005** | **0.074**  **±.021**  **<0.001** | **8.93**  **±0.62**  **NS** | **34**  **±2.32**  **NS** | **Mean**  **±SEM**  **P** |

P: Significance by LSD at P<0.05 from control group.

NS: Not significant.

**Table (5):** Duodenal motility parameters [frequency of contraction (no. of contractions / min.), average duration of contraction (sec.), average force of contraction (gm.) and motility index (gm.min.)] in garlic oil treated diabetic group.

| **Motility index (gm.min.)** | **Average force of contraction**  **(gm.)** | **Average duration of contraction (sec.)** | **Frequency of contraction (/min)** | **Rat**  **No.** |
| --- | --- | --- | --- | --- |
| 2.184 | 0.182 | 12 | 40 | 1 |
| 2.106 | 0.195 | 10.8 | 38 | 2 |
| 2.34 | 0.195 | 12 | 37 | 3 |
| 1.326 | 0.13 | 10.2 | 37 | 4 |
| 0.123 | 0.104 | 10.8 | 35 | 5 |
| 0.585 | 0.065 | 9 | 38 | 6 |
| 1.755 | 0.117 | 15 | 32 | 7 |
| 0.406 | 0.052 | 7.8 | 34 | 8 |
| 1.193 | 0.117 | 10.2 | 35 | 9 |
| **1.335**  **±0.274**  **NS**  **<0.05** | **0.129**  **±.0177**  **NS**  **<0.02** | **10.87**  **±0.68**  **NS**  **<0.05** | **36.22**  **±0.813**  **NS**  **NS** | **Mean**  **±SEM**  **P**  **P*** |

P: Significance by LSD at P<0.05 from control group.

P*: Significance by LSD at P<0.05 from the diabetic group.

NS: Not significant.

**Table (6):** Jejunal motility parameters [frequency of contraction (no. of contractions / min.), average duration of contraction (sec.), average force of contraction (gm.) and motility index (gm.min.)] in control group.

| **Motility index (gm.min.)** | **Average force of contraction**  **(gm.)** | **Average duration of contraction (sec.)** | **Frequency of contraction (/min)** | **Rat**  **No.** |
| --- | --- | --- | --- | --- |
| 1.498 | 0.156 | 9.6 | 37 | 1 |
| 1.755 | 0.195 | 9 | 37 | 2 |
| 1.825 | 0.169 | 10.8 | 41 | 3 |
| 1.115 | 0.143 | 7.8 | 36 | 4 |
| 1.622 | 0.169 | 9.6 | 37 | 5 |
| 1.95 | 0.13 | 15 | 37 | 6 |
| 1.56 | 0.13 | 12 | 35 | 7 |
| 1.544 | 0.143 | 10.8 | 34 | 8 |
| 2.028 | 0.169 | 12 | 38 | 9 |
| **1.655**  **±0.092** | **0.156**  **±.007** | **10.73**  **±0.70** | **36.89**  **±0.66** | **Mean**  **±SEM** |

**Table (7):** Jejunal motility parameters [frequency of contraction (no. of contractions / min.), average duration of contraction (sec.), average force of contraction (gm.) and motility index (gm.min.)] in garlic oil supplemented group.

| **Motility index (gm.min.)** | **Average force of contraction**  **(gm.)** | **Average duration of contraction (sec.)** | **Frequency of contraction (/min)** | **Rat**  **No.** |
| --- | --- | --- | --- | --- |
| 0.281 | 0.026 | 10.8 | 34 | 1 |
| 0.468 | 0.052 | 9 | 34 | 2 |
| 0.374 | 0.039 | 9.6 | 32 | 3 |
| 0.351 | 0.039 | 9 | 36 | 4 |
| 0.265 | 0.026 | 10.2 | 34 | 5 |
| 0.468 | 0.065 | 7.2 | 39 | 6 |
| 0.468 | 0.039 | 12 | 36 | 7 |
| 0.304 | 0.039 | 7.8 | 33 | 8 |
| 0.25 | 0.026 | 9.6 | 34 | 9 |
| **0.359**  **±0.03**  **<0.001** | **0.039**  **±.004**  **<0.001** | **9.467**  **±0.488**  **NS** | **34.67**  **±0.69**  **NS** | **Mean**  **±SEM**  **P** |

P: Significance by LSD at P< 0.05 from control group.

NS: Not significant.

**Table (8):** Jejunal motility parameters [frequency of contraction (no. of contractions / min.), average duration of contraction (sec.), average force of contraction (gm.) and motility index (gm.min.)] in diabetic group.

| **Motility index (gm.min.)** | **Average force of contraction**  **(gm.)** | **Average duration of contraction (sec.)** | **Frequency of contraction (/min)** | **Rat**  **No.** |
| --- | --- | --- | --- | --- |
| 0.562 | 0.078 | 7.2 | 40 | 1 |
| 0.312 | 0.052 | 6 | 32 | 2 |
| 0.257 | 0.039 | 6.6 | 35 | 3 |
| 0.351 | 0.039 | 9 | 34 | 4 |
| 0.281 | 0.026 | 10.8 | 32 | 5 |
| 0.749 | 0.078 | 9.6 | 36 | 6 |
| 0.468 | 0.052 | 9 | 30 | 7 |
| 0.374 | 0.052 | 7.2 | 35 | 8 |
| 0.983 | 0.091 | 10.8 | 38 | 9 |
| **0.482**  **±0.08**  **<0.001** | **0.056**  **±0.007**  **<0.001** | **8.47**  **±0.6**  **<0.05** | **34.67**  **±1.04**  **NS** | **Mean**  **±SEM**  **P** |

P: Significance by LSD at P< 0.05 from control group.

NS: Not significant.

**Table (9):** Jejunal motility parameters [frequency of contraction (no. of contractions / min.), average duration of contraction (sec.), average force of contraction (gm.) and motility index (gm.min.)] in garlic oil treated diabetic group.

| **Motility index (gm.min.)** | **Average force of contraction**  **(gm.)** | **Average duration of contraction (sec.)** | **Frequency of contraction (/min)** | **Rat**  **No.** |
| --- | --- | --- | --- | --- |
| 1.1154 | 0.143 | 7.8 | 38 | 1 |
| 1.56 | 0.13 | 12 | 33 | 2 |
| 2.925 | 0.195 | 15 | 35 | 3 |
| 1.3728 | 0.143 | 9.6 | 38 | 4 |
| 1.56 | 0.104 | 15 | 33 | 5 |
| 0.624 | 0.065 | 9.6 | 38 | 6 |
| 1.092 | 0.091 | 12 | 31 | 7 |
| 0.585 | 0.065 | 9 | 32 | 8 |
| 0.936 | 0.104 | 9 | 33 | 9 |
| **1.308**  **±.024**  **NS**  **<0.001** | **0.116**  **±0.014**  **<0.005**  **<0.001** | **11**  **±0.88**  **NS**  **<0.02** | **34.56**  **±0.93**  **NS**  **NS** | **Mean**  **±SEM**  **P**  **P*** |

P: Significance by LSD at P<0.05 from control group.

P*: Significance by LSD at P<0.05 from the diabetic group.

NS: Not significant.

**Table (10):** Ileal motility parameters [frequency of contraction (no. of contractions / min.), average duration of contraction (sec.), average force of contraction (gm.) and motility index (gm.min.)] in control group.

| **Motility index (gm.min.)** | **Average force of contraction**  **(gm.)** | **Average duration of contraction (sec.)** | **Frequency of contraction (/min)** | **Rat**  **No.** |
| --- | --- | --- | --- | --- |
| 2.5194 | 0.221 | 11.4 | 39 | 1 |
| 2.1216 | 0.208 | 10.2 | 38 | 2 |
| 2.0748 | 0.182 | 11.4 | 34 | 3 |
| 1.3104 | 0.182 | 7.2 | 35 | 4 |
| 1.0296 | 0.143 | 7.2 | 38 | 5 |
| 1.17 | 0.13 | 9 | 37 | 6 |
| 1.482 | 0.13 | 11.4 | 34 | 7 |
| 1.404 | 0.13 | 10.8 | 35 | 8 |
| 1.56 | 0.13 | 12 | 36 | 9 |
| **1.63**  **±0.166** | **0.162**  **±0.012** | **10.07**  **±0.62** | **36.22**  **±0.62** | **Mean**  **±SEM** |

**Table (11):** Ileal motility parameters [frequency of contraction (no. of contractions / min.), average duration of contraction (sec.), average force of contraction (gm.) and motility index (gm.min.)] in garlic supplemented group.

| **Motility index (gm.min.)** | **Average force of contraction**  **(gm.)** | **Average duration of contraction (sec.)** | **Frequency of contraction (/min)** | **Rat**  **No.** |
| --- | --- | --- | --- | --- |
| 0.663 | 0.065 | 10.2 | 33 | 1 |
| 0.585 | 0.065 | 9 | 31 | 2 |
| 0.437 | 0.052 | 8.4 | 32 | 3 |
| 0.33 | 0.039 | 7.8 | 35 | 4 |
| 0.281 | 0.026 | 10.8 | 34 | 5 |
| 0.172 | 0.026 | 6.6 | 41 | 6 |
| 0.281 | 0.026 | 10.8 | 36 | 7 |
| 0.53 | 0.052 | 10.2 | 35 | 8 |
| 0.218 | 0.026 | 8.4 | 33 | 9 |
| **0.389**  **±0.058**  **<0.001** | **0.042**  **±0.006**  **<0.001** | **9.13**  **±0.49**  **NS** | **34.44**  **±0.97**  **NS** | **Mean**  **±SEM**  **P** |

P: Significance by LSD at P< 0.05 from control group.

NS: Not significant.

**Table (12):** Ileal motility parameters [frequency of contraction (no. of contractions / min.), average duration of contraction (sec.), average force of contraction (gm.) and motility index (gm.min.)] in diabetic group.

| **Motility index (gm.min.)** | **Average force of contraction**  **(gm.)** | **Average duration of contraction (sec.)** | **Frequency of contraction (/min)** | **Rat**  **No.** |
| --- | --- | --- | --- | --- |
| 0.702 | 0.078 | 9 | 40 | 1 |
| 0.234 | 0.039 | 6 | 37 | 2 |
| 0.343 | 0.052 | 6.6 | 34 | 3 |
| 0.328 | 0.039 | 8.4 | 33 | 4 |
| 0.25 | 0.026 | 9.6 | 30 | 5 |
| 2.293 | 0.182 | 12.6 | 29 | 6 |
| 0.265 | 0.026 | 10.2 | 30 | 7 |
| 0.218 | 0.026 | 8.4 | 37 | 8 |
| 0.663 | 0.065 | 10.2 | 38 | 9 |
| **0.589**  **±0.222**  **<0.001** | **0.059**  **±.0165**  **<0.001** | **9**  **±0.66**  **NS** | **34.22**  **±1.33**  **NS** | **Mean**  **±SEM**  **P** |

P: Significance by LSD at P< 0.05 from control group.

NS: Not significant.

**Table (13):** Ileal motility parameters [frequency of contraction (no. of contractions / min.), average duration of contraction (sec.), average force of contraction (gm.) and motility index (gm.min.)] in garlic oil treated diabetic group.

| **Motility index (gm.min.)** | **Average force of contraction**  **(gm.)** | **Average duration of contraction (sec.)** | **Frequency of contraction (/min)** | **Rat**  **No.** |
| --- | --- | --- | --- | --- |
| 1.061 | 0.104 | 10.2 | 37 | 1 |
| 1.17 | 0.13 | 9 | 33 | 2 |
| 1.966 | 0.117 | 16.8 | 34 | 3 |
| 1.092 | 0.091 | 12 | 35 | 4 |
| 1.56 | 0.104 | 15 | 34 | 5 |
| 0.624 | 0.065 | 9.6 | 37 | 6 |
| 1.373 | 0.104 | 13.2 | 31 | 7 |
| 0.437 | 0.052 | 8.4 | 32 | 8 |
| 1.193 | 0.117 | 10.2 | 34 | 9 |
| **1.164**  **±0.153**  **<0.05**  **<0.02** | **0.098**  **±0.008**  **<0.001**  **<0.05** | **11.6**  **±0.959**  **NS**  **<0.02** | **34.11**  **±0.676**  **NS**  **NS** | **Mean**  **±SEM**  **P**  **P*** |

P: Significance by LSD at P< 0.05 from control group.

P*: Significance by LSD at P<0.05 from the diabetic group.

NS: Not significant.

**Table (14):** Fasting blood glucose (mg%) in the different studied

groups.

| **Rat number** | **Control group** | **Garlic**  **supplemented group** | **Diabetic group** | **garlic oil treated Diabetic group** |
| --- | --- | --- | --- | --- |
| **1** | 70 | 80 | 254 | 99 |
| **2** | 68 | 83 | 195 | 107 |
| **3** | 72 | 79 | 202 | 212 |
| **4** | 70 | 86 | 169 | 101 |
| **5** | 74 | 76 | 245 | 73 |
| **6** | 80 | 70 | 135 | 102 |
| **7** | 69 | 88 | 129 | 88 |
| **8** | 83 | 70 | 184 | 109 |
| **9** | 77 | 76 | 110 | 90 |
| **Mean**  **±SEM**  **P**  **P*** | **73.67**  **± 1.76** | **78.67**  **± 2.13**  **NS** | **180.33**  **± 16.7**  **<0.001** | **109**  **± 13.4**  **<0.05**  **<0.001** |

P: Significance by LSD at P< 0.05 from control group.

P*: Significance by LSD at P<0.05 from the diabetic group.

NS: Not significant.

**Table (15):** Fasting insulin level (μIU/ml) in the different studied groups.

| **Rat number** | **Control group** | **Garlic treated group** | **Diabetic group** | **Garlic oil treated Diabetic group** |
| --- | --- | --- | --- | --- |
| **1** | 5.07 | 4.57 | 4.93 | 4.55 |
| **2** | 4.35 | 4.21 | 4.82 | 4.91 |
| **3** | 4.66 | 4.28 | 4.99 | 4.53 |
| **4** | 4.84 | 4.38 | 5.31 | 5.13 |
| **5** | 4.57 | 4.13 | 4.31 | 5.07 |
| **6** | 4.64 | 4.75 | 4.6 | 4.92 |
| **7** | 4.84 | 4.66 | 4.72 | 4.67 |
| **8** | - | 4.99 | 5.46 | - |
| **9** | - | 4.87 | 6.03 | - |
| **Mean**  **±SEM**  **P**  **P*** | **4.71**  **±0.09** | **4.54**  **±0.1**  **NS** | **5.02**  **±0.17**  **NS** | **4.83**  **±0.09**  **NS**  **NS** |

P: Significance by LSD at P< 0.05 from control group.

P*: Significance by LSD at P<0.05 from the diabetic group.

NS: Not significant.

**Table (16):** HOMA-IR score in the different studied groups.

| **Rat number** | **Control group** | **Garlic treated group** | **Diabetic group** | **Garlic oil treated Diabetic group** |
| --- | --- | --- | --- | --- |
| **1** | 0.876 | 0.903 | 3.092 | 1.2 |
| **2** | 0.773 | 0.863 | 2.321 | 2.57 |
| **3** | 0.805 | 0.835 | 2.489 | 1.13 |
| **4** | 0.884 | 0.93 | 1.723 | 0.925 |
| **5** | 0.903 | 0.775 | 3.212 | 1.277 |
| **6** | 0.791 | 0.821 | 1.437 | 1.069 |
| **7** | 0.920 | 1.013 | 1.465 | 1.257 |
| **8** | - | 1.706 | 2.144 | - |
| **9** | - | 0.934 | 1.483 | - |
| **Mean**  **±SEM**  **P**  **P*** | **0.85**  **±0.022** | **0.976**  **±0.094**  **NS** | **2.152**  **±0.229**  **<0.001** | **1.35**  **±0.209**  **NS**  **<0.005** |

P: Significance by LSD at P< 0.05 from control group.

P*: Significance by LSD at P<0.05 from the diabetic group.

NS: Not significant.

**Table (17):** HbA1C (gm%) in the different studied groups.

| **Rat number** | **Control group** | **Garlic supplemented group** | **Diabetic group** | **Garlic oil treated Diabetic group** |
| --- | --- | --- | --- | --- |
| 1 | 3.52 | 3.7 | 3.8 | 3.8 |
| 2 | 3.6 | 3.78 | 3.9 | 3.9 |
| 3 | 3.51 | 3.7 | 3.7 | 5.5 |
| 4 | 3.5 | 4 | 4.2 | 4.1 |
| 5 | 3.8 | 3.5 | 4 | 3.5 |
| 6 | 3.2 | 3.9 | 4.4 | 4.2 |
| 7 | - | - | - | - |
| 8 | - | - | - | - |
| 9 | - | - | - | - |
| **Mean**  **±SEM**  **P**  **P*** | **3.52**  **±0.08** | **3.76**  **± 0.07**  **NS** | **4**  **±0.11**  **<0.05** | **4.17**  **±0.29**  **<0.02**  **NS** |

P: Significance by LSD at P< 0.05 from control group.

P*: Significance by LSD at P<0.05 from the diabetic group.

NS: Not significant.

.

**Table (18):** BMI in the different studied groups.

| **Rat Number** | **Control group** | **Garlic oil supplemented group** | **Diabetic group** | **Garlic oil treated Diabetic group** |
| --- | --- | --- | --- | --- |
| 1 | 0.36 | 0.53 | 0.48 | 0.45 |
| 2 | 0.38 | 0.52 | 0.41 | 0.52 |
| 3 | 0.39 | 0.45 | 0.35 | 0.6 |
| 4 | 0.33 | 0.55 | 0.39 | 0.62 |
| 5 | 0.39 | 0.47 | 0.48 | 0.5 |
| 6 | 0.39 | 0.54 | 0.5 | 0.5 |
| 7 | 0.33 | 0.52 | 0.43 | 0.55 |
| 8 | 0.37 | 0.5 | 0.41 | 0.48 |
| 9 | 0.45 | 0.5 | 0.33 | 0.5 |
| **Mean**  **±SEM**  **P**  **P*** | **0.38**  **±0.012** | **0.51**  **±0.011**  **<0.001** | **0.42**  **±0.02**  **NS** | **0.52**  **±0.019**  **<0.001**  **<0.001** |

P: Significance by LSD at P< 0.05 from control group.

P*: Significance by LSD at P<0.05 from the diabetic group.

NS: Not significant.

**Table (19):** Lee index in the different studied groups.

| **Rat number** | **Control group** | **Garlic oil supplemented group** | **Diabetic group** | **Garlic oil treated Diabetic group** |
| --- | --- | --- | --- | --- |
| 1 | 0.258 | 0.299 | 0.285 | 0.274 |
| 2 | 0.264 | 0.297 | 0.294 | 0.291 |
| 3 | 0.263 | 0.280 | 0.251 | 0.291 |
| 4 | 0.246 | 0.304 | 0.263 | 0.314 |
| 5 | 0.255 | 0.314 | 0.267 | 0.289 |
| 6 | 0.263 | 0.319 | 0.297 | 0.294 |
| 7 | 0.246 | 0.291 | 0.278 | 0.298 |
| 8 | 0.257 | 0.287 | 0.283 | 0.283 |
| 9 | 0.282 | 0.282 | 0.246 | 0.287 |
| **Mean**  **±SEM**  **P**  **P*** | **0.259**  **±0.004** | **0.297**  **±0.005**  **<0.001** | **0.274**  **±0.006**  **<0.05** | **0.291**  **±0.004**  **<0.001**  **<0.02** |

P: Significance by LSD at P< 0.05 from control group.

P*: Significance by LSD at P<0.05 from the diabetic group.

**Table (20):** Waist circumference (cm) in the different studied groups.

| **Rat number** | **Control group** | **Garlic supplemented group** | **Diabetic group** | **Garlic oil treated Diabetic group** |
| --- | --- | --- | --- | --- |
| 1 | 12.5 | 13.5 | 14 | 14 |
| 2 | 13 | 14 | 14.5 | 13.5 |
| 3 | 13 | 14 | 13.5 | 13.5 |
| 4 | 12 | 14.5 | 14 | 15 |
| 5 | 12.5 | 15 | 14.5 | 16 |
| 6 | 15 | 13 | 17 | 16.5 |
| 7 | 14 | 16 | 16.5 | 15.5 |
| 8 | 13.5 | 14 | 16 | 14 |
| 9 | 13.5 | 14.5 | 15 | 15 |
| **Mean**  **±SEM**  **P**  **P*** | **13.22**  **±0.30** | **14.28**  **±0.29**  **<0.05** | **15**  **±0.41**  **<0.002** | **14.78**  **±0.36**  **<0.005**  **NS** |

P: Significance by LSD at P< 0.05 from control group.

P*: Significance by LSD at P<0.05 from the diabetic group.

NS: Not significant.

**Table (21):** Duodenal tissue malondialdehyde (MDA, nmol/gm wet tissue) level in the different studied groups.

| **Garlic oil treated Diabetic group** | **Diabetic group** | **Garlic oil supplemented group** | **Control group** | **Rat**  **No.** |
| --- | --- | --- | --- | --- |
| 20.19 | 63.19 | 12.62 | 99.87 | **1** |
| 30.05 | 143.44 | 29.07 | 96.78 | **2** |
| 45.90 | 165.17 | 9.40 | 45.12 | **3** |
| 6.59 | 103.38 | 59.02 | 103.24 | **4** |
| 12.02 | 182.41 | 30.52 | 126.42 | **5** |
| 110.87 | 155.55 | 50.85 | 40.08 | **6** |
| - | 159.21 | 129.57 | - | **7** |
| **37.6**  **±15.72**  **<0.05**  **<0.001** | **138.91**  **±15.65**  **<0.05** | **45.86**  **±15.55**  **<0.05** | **85.25**  **±14.16** | **Mean**  **±SEM**  **P**  **P*** |

P: Significance by LSD at P<0.05 from control group.

P*: Significance by LSD at P<0.05 from the diabetic group.

NS: Not significant.

**Table (22):** Jejunal tissue malondialdehyde (MDA, nmol/gm wet tissue) level in the different studied groups.

| **Garlic oil treated Diabetic group** | **Diabetic group** | **Garlic oil supplemented group** | **Control group** | **Rat**  **No.** |
| --- | --- | --- | --- | --- |
| 115.89 | 406.99 | 142.29 | 25.17 | **1** |
| 199.44 | 338.06 | 38.32 | 97.89 | **2** |
| 230.57 | 240.82 | 238.94 | 44.03 | **3** |
| 537.72 | 186.97 | 73.05 | 134.18 | **4** |
| 186.72 | 311.33 | 51.44 | 113.11 | **5** |
| 364.37 | 218.72 | 32.04 | 171.97 | **6** |
| - | 248.25 | - | - | **7** |
| **272.45**  **±62.65**  **<0.01**  **NS** | **278.73**  **±29.04**  **<0.005** | **96.01**  **±32.92**  **NS** | **97.73**  **±22.53** | **Mean**  **±SEM**  **P**  **P*** |

P: Significance by LSD at P<0.05 from control group.

P*: Significance by LSD at P<0.05 from the diabetic group.

NS: Not significant.

**Table (23):** Ileal tissue malondialdehyde (MDA, nmol/gm wet tissue) level in the different studied groups.

| **Garlic oil treated Diabetic group** | **Diabetic group** | **Garlic oil supplemented group** | **Control group** | **Rat**  **No.** |
| --- | --- | --- | --- | --- |
| 35.86 | 379.19 | 27.04 | 126.18 | **1** |
| 64.05 | 323.27 | 115.08 | 41.39 | **2** |
| 64.11 | 519.37 | 75.52 | 111.07 | **3** |
| 195.72 | 599.04 | 122.30 | 49.19 | **4** |
| 249.66 | 487.10 | 149.85 | 63.33 | **5** |
| 227.71 | 521.79 | 106.97 | 160 | **6** |
| - | 506.56 | - | - | **7** |
| **160.25**  **±40.19**  **NS**  **<0.001** | **476.62**  **±35.48**  **<0.001** | **99.46**  **±17.49**  **NS** | **91.86**  **±19.47** | **Mean**  **±SEM**  **P**  **P*** |

P: Significance by LSD at P<0.05 from control group.

P*: Significance by LSD at P<0.05 from the diabetic group.

NS: Not significant.

**Table (24):** Duodenal tissue glutathione peroxidase (GSH-PX) (U/gm wet tissue) in the different studied groups.

| **Garlic oil treated Diabetic group** | **Diabetic group** | **Garlic oil supplemented group** | **Control group** | **Rat**  **No.** |
| --- | --- | --- | --- | --- |
| 928.05 | 2992.92 | 972.7 | 1367.49 | **1** |
| 193.51 | 722.57 | 1311.46 | 405.25 | **2** |
| 996.52 | 1115.87 | 642.26 | 269.94 | **3** |
| 200.41 | 631.87 | 790.03 | 873.65 | **4** |
| 460.99 | 1230.07 | 1190.02 | 760.14 | **5** |
| 521.58 | 2137.73 | 817.31 | 2026.46 | **6** |
| **550.18**  **±141.46**  **NS**  **<0.02** | **1471.8**  **±374.58**  **NS** | **953.96**  **±104.34**  **NS** | **950.49**  **±266.72** | **Mean**  **±SEM**  **P**  **P*** |

P: Significance by LSD at P<0.05 from control group.

P*: Significance by LSD at P<0.05 from the diabetic group.

NS: Not significant.

**Table (25):** Jejunal tissue glutathione peroxidase (GSH-PX) (U/ gm wet tissue) in the different studied groups.

| **Garlic oil treated Diabetic group** | **Diabetic group** | **Garlic oil supplemented group** | **Control group** | **Rat**  **No.** |
| --- | --- | --- | --- | --- |
| 360.28 | 2024.38 | 406.61 | 778.13 | **1** |
| 400.64 | 2691.51 | 1029.37 | 1414.73 | **2** |
| 448.62 | 2309.05 | 1131.01 | 385.95 | **3** |
| 287.54 | 1570.93 | 821.35 | 3967.92 | **4** |
| 867.16 | 1550.91 | 436.93 | 3076.09 | **5** |
| 938.60 | 480.53 | 260.05 | 1924.6 | **6** |
| **550.47**  **±113.88**  **<0.01**  **<0.02** | **1771.2**  **±313.84**  **NS** | **680.89**  **±147.83**  **<0.02** | **1924.6**  **±561.04** | **Mean**  **±SEM**  **P**  **P*** |

P: Significance by LSD at P<0.05 from control group.

P*: Significance by LSD at P<0.05 from the diabetic group.

NS: Not significant.

**Table (26):** Ileal tissue glutathione peroxidase (GSH-PX) (U/gm wet tissue) in the different studied groups.

| **Garlic oil treated Diabetic group** | **Diabetic group** | **Garlic oil supplemented group** | **Control group** | **Rat**  **No.** |
| --- | --- | --- | --- | --- |
| 745.02 | 2506.89 | 1537.81 | 1267.65 | **1** |
| 329.56 | 1636.08 | 852.15 | 1517.66 | **2** |
| 668.69 | 653.28 | 607.35 | 543.96 | **3** |
| 389.04 | 739.47 | 1132.95 | 273.22 | **4** |
| 1191.85 | 3110.87 | 1087.83 | 705.68 | **5** |
| 1339.33 | 1129.73 | 959.87 | 917.66 | **6** |
| 1305.56 | 876.97 | - | - | **7** |
| **852.72**  **±161.17**  **NS**  **<0.05** | **1629.4**  **±406.58**  **<0.05** | **1015.8**  **±129.65**  **NS** | **878.01**  **±189.27** | **Mean**  **±SEM**  **P**  **P*** |

P: Significance by LSD at P<0.05 from control group.

P*: Significance by LSD at P<0.05 from the diabetic group.

NS: Not significant.

**Table (27):** Duodenal tissue catalase (CAT) activity (U/gm wet tissue) in the different studied groups.

| **Garlic oil treated Diabetic group** | **Diabetic group** | **Garlic oil supplemented group** | **Control group** | **Rat**  **No.** |
| --- | --- | --- | --- | --- |
| 13.55 | 6.66 | 6.55 | 13.29 | **1** |
| 13.71 | 7.86 | 4.21 | 5.95 | **2** |
| 7.98 | 14.62 | 6.93 | 3.31 | **3** |
| 10.42 | 8.79 | 6.54 | 10 | **4** |
| 10.98 | 10.41 | 5.33 | 6.08 | **5** |
| 14.51 | 17.28 | 4.62 | 2.86 | **6** |
| - | - | 8.12 | 5.09 | **7** |
| **11.86**  **±1.02**  **<0.01**  **NS** | **10.94**  **±1.7**  **<0.05** | **6.04**  **±0.52**  **NS** | **6.65**  **±1.42** | **Mean**  **±SEM**  **P**  **P*** |

P: Significance by LSD at P<0.05 from control group.

P*: Significance by LSD at P<0.05 from the diabetic group.

NS: Not significant.

**Table (28):** Jejunal tissue catalase (CAT) activity (U/gm wet tissue) in the different studied groups.

| **Garlic oil treated Diabetic group** | **Diabetic group** | **Garlic oil supplemented group** | **Control group** | **Rat**  **No.** |
| --- | --- | --- | --- | --- |
| 3.53 | 5.95 | 2.54 | 4.52 | **1** |
| 4.49 | 10.76 | 4.99 | 3.22 | **2** |
| 1.69 | 14.26 | 4.05 | 1.63 | **3** |
| 1.16 | 4.22 | 3.12 | 2.74 | **4** |
| 5.27 | 3.71 | 1.03 | 3.95 | **5** |
| 6.5 | 4.36 | 2.52 | 2.04 | **6** |
| - | 5.24 | - | 2.53 | **7** |
| **3.77**  **±0.85**  **NS**  **<0.05** | **6.93**  **±1.52**  **<0.01** | **3.04**  **±0.56**  **NS** | **2.95**  **±0.38** | **Mean**  **±SEM**  **P**  **P*** |

P: Significance by LSD at P<0.05 from control group.

P*: Significance by LSD at P<0.05 from the diabetic group.

NS: Not significant.

**Table (29):** Ileal tissue catalase (CAT) activity (U/gm wet tissue) in the different studied groups.

| **Garlic oil treated Diabetic group** | **Diabetic group** | **Garlic oil supplemented group** | **Control group** | **Rat**  **No.** |
| --- | --- | --- | --- | --- |
| 4.19 | 4.23 | 2.1 | 2.24 | **1** |
| 1.24 | 6.04 | 1.99 | 2.26 | **2** |
| 2.24 | 3.40 | 2.35 | 4.05 | **3** |
| 1.90 | 8.53 | 1.46 | 5.19 | **4** |
| 3.39 | 3.83 | 1.12 | 1.31 | **5** |
| 0.96 | 4.01 | 2.33 | 2.74 | **6** |
| **2.32**  **±0.51**  **NS**  **<0.005** | **5**  **±0.8**  **<0.02** | **1.9**  **±0.2**  **NS** | **2.97**  **±0.58** | **Mean**  **±SEM**  **P**  **P*** |

P: Significance by LSD at P<0.05 from control group.

P*: Significance by LSD at P<0.05 from the diabetic group.

NS: Not significant.
